# A Facile Synthesis of Functionalized Dispirooxindole Derivatives via a Three-Component 1,3-Dipolar Cycloaddition Reaction

**DOI:** 10.3390/molecules18055142

**Published:** 2013-05-03

**Authors:** Jun He, Guang Ouyang, Zhixiang Yuan, Rongsheng Tong, Jianyou Shi, Liang Ouyang

**Affiliations:** 1 State Key Laboratory of Biotherapy, West China Hospital, Sichuan University, Chengdu 610041, China; 2 Sichuan Academy of Medical Sciences, Sichuan Provincial People’s Hospital, Chengdu 610072, China; 3 Institute of Pharmacy, Sichuan Academy of Chinese Medicine Sciences, Chengdu 610041, China

**Keywords:** multicomponent reactions, 1,3-dipolarcycloaddition, azomethine ylide, dispirooxindole

## Abstract

An efficient synthesis of novel dispirooxindoles has been achieved through three-component 1,3-dipolar cycloaddition of azomethine ylides generated *in situ* by the decarboxylative condensation of isatin and an α-amino acid with the dipolarophile 5-benzylideneimidazolidine-2,4-dione**.** The improved procedure features mild reaction conditions, high yields, high diastereoselectivities, a one-pot procedure and operational simplicity.

## 1. Introduction

Nowadays, conventional one-pot, multicomponent reactions (MCRs) are considered to be one of the most efficient strategies in organic and medical chemistry for synthesizing structurally diverse compounds and biologically active natural products, usually in a stereoselective-manner [1]. The highly effective one-pot procedure of MCRs exhibits many advantages, including atom economy, facile synthesis, convergence, productivity and easy execution [2]. MCRs were a common method for building molecular diversities with complex scaffolds and had broad applications in combinatorial chemistry, which allowed a rapid access to identify a promising lead molecule in drug candidate discovery [3,4,5].

The 1,3-dipolar cycloaddition of azomethine ylides with olefinic and acetylenic dipolarophiles is one of the most useful MCRs for providing an useful approach to building nitrogen-containing five-membered ring heterocycles, such as pyrroline, pyrrolidine, pyrrolizidine or spirooxindole derivatives [6,7,8,9], which served as useful molecular scaffolds for the exploration and exploitation of pharmacophore space via diversity-oriented synthesis [10,11,12,13]. Among of them, dispirooxindole ring systems possess more interesting structural properties and have been reported to exhibit strong bioactivity profiles including antimicrobial [14], antitumoral [15], anti-inflammatory [16], anti-HIV [17] and potent non-peptide inhibition of the p53–MDM2 interaction [18].

Hydantoin derivatives are widely used in malignant hyperthermia, neuroleptic malignant syndrome, spasticity, and anticonvulsants [19,20,21,22], especially the spirohydantoins, which are considered to be a novel aldose reduetase inhibitor to treat for diabetes [23]. Significant efforts have been focused on developing a general synthetic route to access those compounds. However, to the best of our knowledge, only a few methods were reported to synthesize the spiropyrrolidine bisoxindoles [24,25], and a general method to prepare dispirooxindole hydantoin derivatives is still lacking. Thus, in order to extend our interest in cycloaddition reactions of novel spiro compounds and nitrogen heterocycles with biological activities [26,27,28], we report herein the efficient synthesis in excellent yields of a series of novel dispirooxindole derivatives by the three-component 1,3-dipolar cycloaddition reaction of nonstabilized azomethine ylides generated *in situ* by the decarboxylative condensation of isatin and primary α-amino acid with the Knoevenagel adduct derivatives (preformed by reaction of hydantoin with substituted benzaldehydes).

## 2. Results and Discussion

**Scheme 1:**
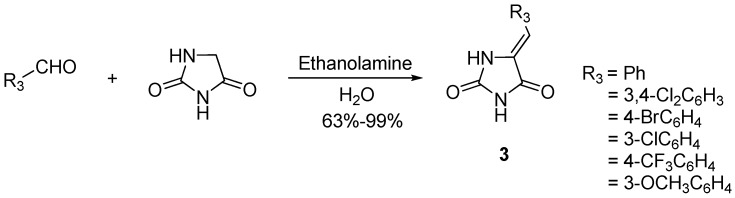
The synthetic route to compounds **3**.

In our initial endeavor, the Knoevenagel adducts **3** were synthesized via a method involving condensation of commercially available hydantoin and substituted benzaldehydes in water using ethanolamine as catalyst. After work-up, the crude reaction mixtures were purified by recrystallization in ethanol/water (40:60) to afford 63-99% total yields of the target products (Scheme 1). Melting point, NMR and mass spectrometry data were consistent with those reported in the literature [29].

**Scheme 2:**
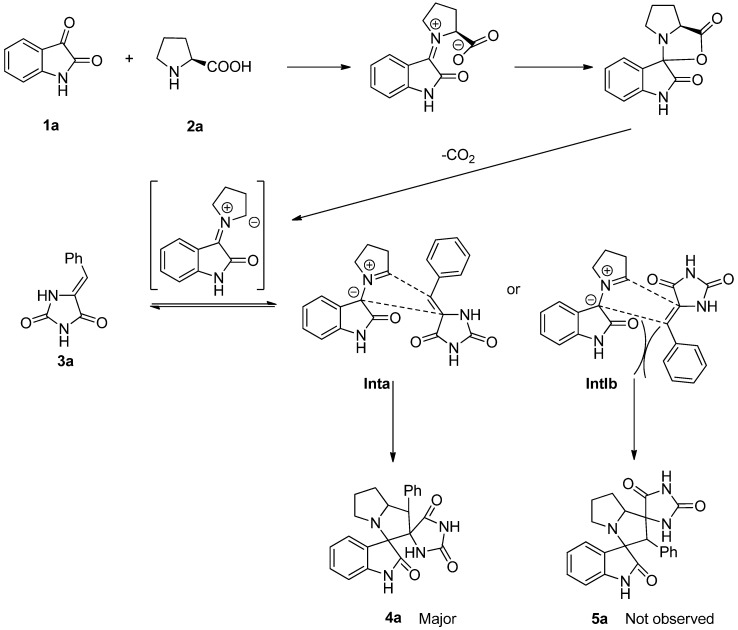
Possible reaction mechanism for the synthesis of dispirooxindole hydantoin derivatives.

From the mechanistic perspective, the azomethine ylides, a powerful class of reagents, have featured in a number of 1,3-dipolar cycloaddition reactions. In combination with the experiences from our previous work, we envisaged that an azomethine ylide could be generated in situ from isatin (**1a**) and L-proline (**2a**), and then trapped with Knoevenagel adduct 5-benzylideneimidazolidine-2,4-dione (**3a**) acting as dipolarophile, to afford spiropyrrolizidine oxindole **4a**. Hence, the 1,3-dipolar cycloaddition reaction would be facilitated in one-pot with two steps. Although the azomethine ylides, generated from the reaction of isatin and L-proline, have two nucleophilic carbons potentially resulting in two regioisomers. However, high regioselectivity was observed in the formation of the product (see below). It may resulted from the more stable transition state (**Inta**) leading to the observed products (**4a)**. Meanwhile, the other possible one (**Intb**) would be less stable because of steric interactions between the aryl ring of Knoevenagel adducts and isatin backbone (Scheme 2).

In an effort to identify the reaction parameters of this one-pot process, the three-component reaction of isatin, L-proline and 5-benzylideneimidazolidine-2,4-dione was carried out as model reaction. Firstly, various solvents were examined under 80 °C，and the results were summarized in Table 1. Acetonitrile and ether solvents such as dioxane and THF gave moderate yields (entries 1–3), and with toluene as solvent, a poor yield resulted (entry 4). To our delight, the alcohol solvent methanol gave promising yields, and ethanol afforded even better results (entries 5–6). Ethanol may facilitate the production of the azomethine ylide by accelerating the formation of an iminium species between isatin and L-proline. Then the reaction temperature, mixed solvent and time were further investigated, the desired product was finally obtained as a single regioisomer in almost quantitative yield (95%) after 10 hours at 50 °C (entries 7–9). Consequently, we chose these conditions for the rest of our studies.

**Table 1:** Optimization of reaction conditions ^a^.

Entry	Solvent	Temp (°C)	Yield ^b^ (%)
1	1,4-dioxane	80	33
2	THF	80	42
3	CH_3_CN	80	31
4	Toluene	80	17
5	Methanol	reflux	57
6	Ethanol	80	68
7	Ethanol/H_2_O	100	55
8	Ethanol	50	74
9 ^c^	Ethanol	50	95

^a^ Unless indicated otherwise, the reaction was performed with **3a** (0.5 mmol), **1a** isatin (0.5 mmol), and L-proline (0.5 mmol) in different solvents (10.0 mL) and temperatures for 5 h. ^b^ Isolated yield based on isatin. ^c^ 10 h.

With the optimized reaction conditions in hand, various structurally diverse 5-benzyli- deneimidazolidine-2,4-diones **3** were investigated. Gratifyingly, the corresponding spiro-pyrrolidine products **4a–4m** were obtained as single diastereoisomers in high yields. As shown in Table 2, different substitutents on the aryl ring, such as bromo, chloro, CF_3_ and OCH_3_ groups at the *meta* or *para* positons all gave corresponding products **4e**, **4i**, **4l** and **4m** in high yields ranging from 85% to 90% (entries 5, 9, 12 and 13). Furthermore, different substituents in the isatin such as 5-F, -Cl or -Br also reacted smoothly to generate the desired products in high yields (entries 3, 6, 7 and 10). More interestingly, the unprotected isatin gave even better results (entry 1 *vs.* 2), which opens a door to further functionalize the products in future medicinal chemistry studies.

**Scheme 3:**
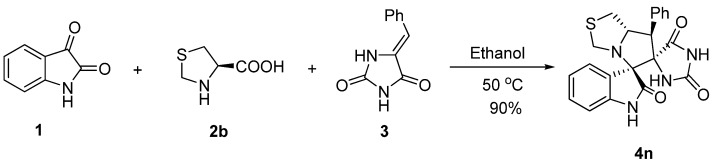
MCRs of thioproline **2b**.

We also studied the cycloaddition reaction of the different amino acid thioproline **2b**, wbereby the reaction between isatin **1**, **2b** and **3** happened smoothly to afford the desired spirothiopyrrolidine **4n** as a single diastereoisomer in 90% yield (Scheme 3).

**Table 2:** Scope of the reaction. 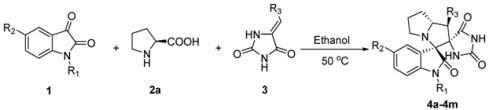

Entry	1	3	4	Yield (%)
1	R_1_ = R_2_ = H	R_3_ = Ph	**4a**	93
2	R_1_ = CH_2_C_6_H_4_, R_2_ = H	R_3_ = Ph	**4b**	89
3	R_1_ = H, R_2_ = Br	R_3_ = Ph	**4c**	92
4	R_1_ = CH_2_C_6_H_4_, R_2_ = H	R_3_ = 3,4-Cl_2_C_6_H_3_	**4d**	83
	R_1_ = R_2_ = H			
5	R_1_ = H, R_2_ = F	R_3_ = 4-BrC6H4	**4e**	90
6	R_1_ = H, R_2_ = Cl	R_3_ = Ph	**4f**	91
7	R_1_ = CH_3_, R_2_ = H	R_3_ = Ph	**4g**	84
8	R_1_ = R_2_ = H	R_3_ = Ph	**4h**	95
9	R_1_ = H, R_2_ = Cl	R_3_ = 3-ClC_6_H_4_	**4i**	88
10	R_1_ = CH_2_C_6_H_4_, R_2_ = H	R_3_ = 3-ClC_6_H_4_	**4j**	93
11	R_1_ = H R_2_ = H	R_3_ = 3-ClC_6_H_4_	**4k**	89
	R_1_ = H R_2_ = H			
12		R_3_ = 4-CF_3_C6H4	**4l**	85
13		R_3_ = 4-OCH_3_C6H4	**4m**	82

**Figure 1:**
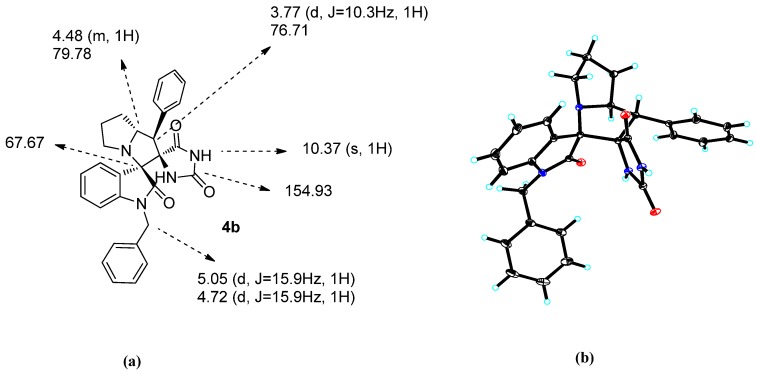
(**a**) Selected ^1^H- and ^13^C-NMR chemical shifts of **4b**. (**b**) Single crystal X-ray diffraction study of compound **4b**.

To further confirm the structure, diastereoselectivity and regioselectivity, detailed NMR spectral and X-ray analyses were carried out. The structures proposed for all products were in agreement with their NMR spectra, as discussed for compound **4b** as an example In the ^1^H-NMR spectrum of **4b**, the pyrrolidine ring proton of C-5 exhibited a multiplet *(m)* peak at δ 4.48 (m, 1H). The C-4 proton which was attached to the aryl group appeared as a doublet at δ 3.77 (d, *J* = 10.3 Hz, 1H). The aromatic protons were distributed in the δ 7.41–6.24 region. The NH proton appeared as a singlet at δ 8.37 and δ 10.37. Based on the calculation of the coupling constant (*J*-based configuration analysis, J > 10 Hz), the relative configuration of this structure should be as same as compound **4b** shown in Figure 1 and the configuration was further confirmed by the X-ray study of a single crystal of compound **4b** (Figure 1b). The results revealed that the pyrrolidine ring adopted an envelope form with the spiro carbon being out of plane. The ^13^C-NMR of compound **4b** supported the proposed structure as well. The pyrrolidine ring carbons resonated in the δ 67.67 ppm region. The carbonyl carbon resonated at δ 154.93 ppm, respectively.

**Scheme 4:**
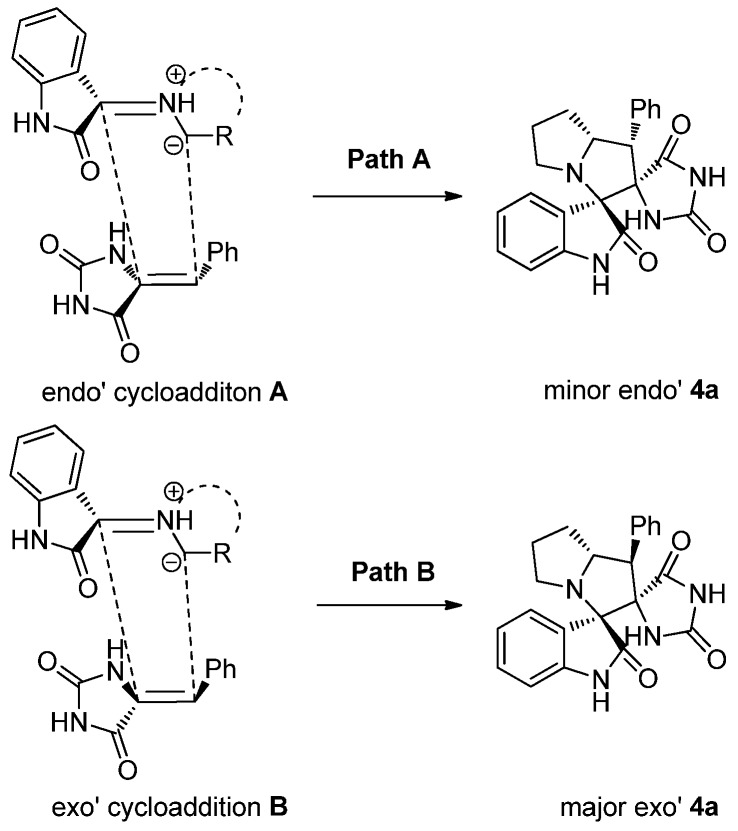
Plausible mechanism for the formation of compound **4**.

The regioselectivity in formation of **4** can be explained by considering the secondary orbital interaction (SOI) mechanism proposed in Scheme 4 [30]. The reaction proceeds through the generation of azomethine ylide via the condensation of isatin with L-proline and decarboxylation. The dipolarophile **3** regioselectively reacts with azomethine ylides in ethanol to give the desired products compounds **4**. The X-ray structure of the product **4b** reflects that the cycloaddition proceeds via an exo’-transition state (Scheme 4, path B). This can be explained by the fact that the corresponding endo’-transition state (**A**) would require more free energy of activation than the exo’-transition state (**B**) leading to **4a** as the former would result in electrostatic repulsion between the *cis* carbonyls increasing the free energy of activation. Accordingly, the observed regioisomer **4** via path B is more favorable because of the SOI which is not possible in path A [31].

## 3. Experimental

### 3.1. General

All reagents were purchased from commercial sources and used without further purification. Melting points are corrected. ^1^H-NMR spectra were determined on a Bruker Avance III 400MHz spectrometer in DMSO-d^6^ solution. J values are in Hz. Chemical shifts are expressed in ppm downfield from internal standard TMS. HRMS data were obtained using Bruker micrOTOF-Q instrument or TOF-MS instrument. The starting compounds **3** were prepared according to the previously reported procedures.

### 3.2. General Procedure for the Synthesis of Dispirooxindoles **4**

A dry 50 mL flask was charged with istain derivatives **1**(0.5 mmol), L-proline, L-thioproline **2a** or **2b** (0.5 mmol), and imidazolidin-2-one derivatives **3** (0.5 mmol), and ethanol (10 mL). The mixture was stirred at 50 °C for 10 h. After completion of the reaction (monitored by TLC), the solvent was cooled, then was filtrated and washed by 10 mL of ethanol twice to give solid. The solid was dried at 80 °C for 4h under vacuum to give compounds **4**. The structures of the products were identified by ^1^H-NMR, ^13^C{1H}-NMR and HRMS spectra. The structure and regiochemistry of the products were assigned on the basis of their spectroscopic analysis.

*1-Phenylhexahydro-1H-pyrrolizine-2-spiro-5'-imidazolidine-2',4'-dione-3-spiro-3''-indoline-2''-one* (**4a**). White solid; m.p. 196–198 °C; ^1^H-NMR (DMSO-d_6_): δ (ppm) 1.56–1.69 (m, 1H, CH_2_), 1.73–2.06 (m, 3H, CH_2_), 2.81–2.95 (m, 1H, CH_2_), 3.70 (d, *J =* 10.3 Hz, 1H, CH), 4.31–4.52 (m, 1H, CH), 6.80 (d, *J* = 7.6 Hz, 1H, ArH), 6.98–7.07 (m, 1H, ArH), 7.19–7.34 (m, 4H, ArH), 7.36 (d, *J* = 7.1 Hz, 2H, ArH), 7.50 (d, *J* = 7.5 Hz, 1H, ArH), 8.10 (s, 1H, NH), 10.33 (s, 1H, NH), 10.42 (s, 1H, NH); ^13^C{1H}-NMR (DMSO-d_6_): δ 27.19, 29.56, 46.43, 55.99, 56.79, 67.50, 76.79, 79.47, 109.78, 120.98, 124.21, 127.36, 128.20, 128.32, 129.31, 129.66, 134.80, 154.93, 172.39, 176.38; HRMS: calcd. for C_22_H_20_N_4_O_3_^+^ [M+H]^+^: 389.1613, found: 389.1614. 

*1-Phenylhexahydro-1H-pyrrolizine-2-spiro-5'-imidazolidine-2',4'-dione-3-spiro-3''-N-benzylindoline-2''-one* (**4b**). White solid; m.p. 187–189 °C; ^1^H-NMR (DMSO-d_6_): δ (ppm) 1.51–1.73 (m, 1H, CH_2_), 1.75–2.10 (m, 3H, CH_2_), 2.81–2.92 (m, 1H, CH_2_). 3.77 (d, *J* = 10.3 Hz, 1H, CH), 4.33–4.41 (brs, 1H, CH_2_), 4.43–4.53 (m, 1H, CH), 4.72 (d, *J* = 15.9 Hz, 1H, CH_2_), 5.05 (d, *J* = 15.9 Hz, 1H, CH_2_), 6.82 (d, *J* = 7.7 Hz, 1H, ArH), 7.01–7.14 (m, 1H, ArH), 7.18–7.35 (m, 9H, ArH), 7.36–7.46 (d, *J* = 6.9 Hz, 2H, ArH), 7.59 (d, *J* = 7.2 Hz, 1H, ArH), 8.37 (s, 1H, NH), 10.37 (s, 1H, NH); ^13^C{1H}-NMR (DMSO-d_6_): δ 27.47, 29.67, 42.75, 46.49, 56.06, 67.67, 76.71, 79.78, 109.35, 121.77, 123.69, 126.96, 127.35, 127.51, 128.28, 128.31, 129.4, 129.78, 134.67, 136.12, 142.46, 154.93, 172.15, 174.95; HRMS: calcd. for C_29_H_26_N_4_O_3_^+^ [M+H]^+^: 479.2080, found: 479.2083. 

*1-Phenylhexahydro-1H-pyrrolizine-2-spiro-5'-imidazolidine-2',4'-dione-3-spiro-3''-5-bromoindoline-2''-one* (**4c**). White solid; m.p. 175–177 °C; ^1^H-NMR (DMSO-d_6_): δ (ppm) 1.57–1.70 (m, 1H, CH_2_), 1.75–2.08 (m, 3H, CH_2_), 2.73–2.90 (m, 1H, CH_2_ ), 3.66 (d, *J* = 10.5 Hz, 1H, CH), 4.34–4.46 (m, 1H, CH), 6.75 (d, *J* = 8.3 Hz, 1H, ArH), 7.23–7.34 (m, 3H, ArH), 7.37 (d, *J* = 7.0 Hz, 2H, ArH), 7.45 (dd, *J* = 8.3, 1.5 Hz, 1H, ArH), 7.60 (s, 1H, ArH), 8.29 (s, 1H, NH), 10.44 (s, 1H, NH), 10.59 (s, 1H, NH); ^13^C{1H}-NMR (DMSO-d_6_): δ 27.35, 29.52, 46.45, 56.74, 67.50, 76.87, 79.65, 111.79, 112.98, 126.70, 127.48, 128.24, 129.71, 130.67, 132.28, 134.55, 141.52, 154.85, 172.34, 175.93; HRMS: calcd. for C_22_H_19_BrN_4_O_3_^+^ [M+H]^+^: 467.0728, found: 467.0719.

*1-(3,4-Dichloro)phenylhexahydro-1H-pyrrolizine-2-spiro-5'-imidazolidine-2',4'-dione-3-spiro-3''-N-benzylindoline-2''-one* (**4d**). White solid; m.p. 160–162 °C; ^1^H-NMR (DMSO-d_6_): δ (ppm) 1.53–1.67 (m, 1H, CH_2_), 1.68-1.80 (m, 1H, CH_2_), 1.95–2.11 (m, 2H, CH_2_), 2.62 (t, *J* = 7.2 Hz, 1H, CH_2_), 3.45–3.55 (m, 1H, CH_2_), 4.25 (d, *J* = 8.0 Hz, 1H, CH), 4.66–4.79 (m, 2H, 1/2CH_2_, CH), 5.05 (d, *J* = 15.7 Hz, 1H, CH_2_), 6.84 (d, *J* = 7.8 Hz, 1H, ArH), 7.01–7.10 (m, 1H, ArH), 7.21–7.31 (m, 4H, ArH), 7.34 (d, *J* = 7.1 Hz, 2H, ArH), 7.53 (dd, *J* = 8.4, 2.3 Hz, 2H, ArH), 7.63 (d, *J* = 2.2 Hz, 1H, ArH), 7.87 (s, 1H, ArH), 7.98 (d, *J* = 8.6 Hz, 1H, ArH), 10.68 (s, 1H, NH); ^13^C{1H}-NMR (DMSO-d_6_): δ 25.05, 29.22, 42.62, 47.58, 53.38, 68.27, 76.75, 78.47, 109.57, 122.27, 123.31, 127.42, 127.84, 128.47, 128.64, 130.04, 132.49, 132.63, 133.20, 135.48, 135.83, 143.65, 155.35, 174.80, 175.40; HRMS: calcd. for C_29_H_24_Cl_2_N_4_O_3_^+^ [M+H]^+^: 547.1300, found: 547.1304.

*1-(4-Bromo)phenylhexahydro-1H-pyrrolizine-2-spiro-5'-imidazolidine-2',4'-dione-3-spiro-3''-indoline-2''-one* (**4e**). White solid; m.p. 222–224 °C; ^1^H-NMR (DMSO-d_6_): δ (ppm) 1.59–1.70 (m, 1H, CH_2_), 1.72–2.05 (m, 3H, CH_2_), 2.83–2.96 (m, 1H, CH_2_), 3.70 (d, *J* = 10.3 Hz, 1H, CH), 4.32–4.40 (m, 1H, CH), 6.80 (d, *J* = 7.6 Hz, 1H, ArH), 6.97–7.06 (m, 1H, ArH), 7.19–7.28 (m, 1H, ArH), 7.33 (d, *J* = 8.5 Hz, 2H, ArH), 7.43–7.59 (m, 3H, ArH), 8.21 (s, 1H, NH), 10.41 (s, 1H, NH), 10.45 (s, 1H, NH); ^13^C{1H}-NMR (DMSO-d_6_): δ 27.13, 29.46, 46.52, 56.26, 67.60, 76.78, 79.40, 109.86, 120.86, 121.06, 124.13, 128.37, 129.44, 131.16, 131.92, 134.34, 142.22, 155.01, 172.46, 176.34; HRMS: calcd. for C_22_H_19_BrN_4_O_3_^+^ [M+H]^+^: 467.0718, found: 467.0719.

*1-Phenylhexahydro-1H-pyrrolizine-2-spiro-5'-imidazolidine-2',4'-dione-3-spiro-3''-5-fluoroindoline-2''-one* (**4f**). White solid; m.p. 196–197 °C; ^1^H-NMR (DMSO-d_6_): δ (ppm) 1.59–1.74 (m, 1H, CH_2_), 1.76–1.94 (m, 2H, CH_2_), 1.95–2.071 (m, 1H, CH_2_), 2.72–2.83 (m, 1H, CH_2_), 3.71 (d, *J* = 10.4 Hz, 1H, CH), 4.33–4.43 (m, 1H, CH), 6.79 (dd, *J* = 8.5, 4.6 Hz, 1H, ArH), 7.06–7.14 (m, 1H, ArH), 7.22–7.33 (m, 3H, ArH), 7.34–7.43 (m, 3H, ArH), 8.35 (s, 1H, NH), 10.37 (s, 1H, NH), 10.43 (s, 1H, NH); ^13^C{1H}-NMR (DMSO-d_6_): δ 27.74, 29.63, 46.26, 56.35, 67.52, 77.11, 79.72, 110.45, 110.53, 115.71, 115.95, 116.21, 126.02, 126.10, 127.48, 128.19, 129.73, 134.60, 138.25, 154.89, 156.72, 158.62, 171.89, 176.34; HRMS: calcd. for C_22_H_19_FN_4_O_3_^+^ [M+H]^+^: 407.1520, found: 407.1519.

*1-Phenylhexahydro-1H-pyrrolizine-2-spiro-5'-imidazolidine-2',4'-dione-3-spiro-3''-5-chloroindoline-2''-one* (**4g**). White solid; m.p. 193–195 °C; ^1^H-NMR (DMSO-d_6_): δ (ppm) 1.56–1.71 (m, 1H, CH_2_), 1.76–1.95 (m, 2H, CH_2_), 1.96–2.06 (m, 1H, CH_2_), 2.76–2.87(m, 1H, CH_2_), 3.67 (d, *J* = 10.4 Hz, 1H, CH), 4.32–4.44 (m, 1H, CH), 6.82 (d, *J* = 8.3 Hz, 1H, ArH), 7.22–7.34 (m, 4H, ArH), 7.33–7.41 (m, 2H, ArH), 7.49 (d, *J* = 1.9 Hz, 1H, ArH), 8.30 (s, 1H, NH), 10.42 (s, 1H, NH), 10.57 (s, 1H, NH); ^13^C{1H}-NMR (DMSO-d_6_): δ 27.50, 29.58, 46.42, 56.67, 67.53, 76.94, 79.70, 111.29, 125.25, 126.33, 127.51, 128.09, 128.25, 129.43, 129.73, 134.56, 141.09, 154.89, 172.24, 176.10; HRMS: calcd. for C_22_H_19_ClN_4_O_3_^+^ [M+H]^+^: 423.1225, found: 423.1224.

*1-Phenylhexahydro-1H-pyrrolizine-2-spiro-5'-imidazolidine-2',4'-dione-3-spiro-3''-N-methylindoline-2''-one* (**4h**). White solid; m.p. 199–201 °C; ^1^H-NMR (DMSO-d_6_): δ (ppm) 1.58–1.71 (m, 1H, CH_2_), 1.73–2.09 (m, 3H, CH_2_), 2.80–2.93 (m, 1H, CH_2_), 3.10 (s, 3H,CH_3_), 3.74 (d, *J* = 10.4 Hz, 1H, CH), 4.38–4.50 (m, 1H, CH), 7.00 (d, *J* = 7.8 Hz, 1H, ArH), 7.06–7.16 (m, 1H, ArH), 7.22–7.43 (m, 6H, ArH), 7.57 (d, *J* = 7.5 Hz, 1H, ArH), 8.18 (s, 1H, NH), 10.34 (s, 1H, NH), ^13^C{1H}-NMR (DMSO-d_6_): δ 26.29, 27.28, 29.61, 46.53, 57.04, 67.59, 76.57, 79.58, 108.79, 121.74, 123.65, 127.48, 128.03, 128.28, 129.54, 129.74, 134.78, 143.63, 154.92, 172.40, 174.69; HRMS: calcd. for C_23_H_22_N_4_O_3_^+^ [M+H]^+^: 403.1767, found: 403.1770.

*1-(3-Chloro)phenylhexahydro-1H-pyrrolizine-2-spiro-5'-imidazolidine-2',4'-dione-3-spiro-3''-indoline-2''-one* (**4i**). White solid; m.p. 156–128 °C; ^1^H-NMR (DMSO-d_6_): δ (ppm) 1.58–1.71 (m, 1H, CH_2_), 1.74–7.97 (m, 3H, CH_2_), 2.79–2.91 (m, 1H, CH_2_), 3.74 (d, *J* = 10.3 Hz, 1H, CH), 4.31–4.44 (m, 1H, CH), 6.80 (d, *J* = 7.7 Hz, 1H, ArH), 6.95–7.07 (m, 1H, ArH), 7.15–7.28 (m, 1H, ArH), 7.29–7.40 (brs, 3H, ArH), 7.45 (s, 1H, ArH), 7.52 (d, *J* = 7.5 Hz, 1H, ArH), 8.32 (s, 1H, NH), 10.40 (s, 1H, NH), 10.43 (s, 1H, NH); ^13^C{1H}-NMR (DMSO-d_6_): δ 27.29, 29.48, 46.44, 56.19, 67.57, 76.76, 79.47, 109.85, 121.03, 124.07, 127.50, 128.46, 129.40, 129.49,130.02, 132.89, 137.46, 142.13, 154.98, 172.21, 176.27; HRMS: calcd. for C_22_H_19_ClN_4_O_3_^+^ [M+H]^+^: 423.1224, found: 423.1224. 

*1-(3-Chloro)phenylhexahydro-1H-pyrrolizine-2-spiro-5'-imidazolidine-2',4'-dione-3-spiro-3''-5-chloroindoline-2''-one* (**4j**). White solid; m.p. 161–163 °C; ^1^H-NMR (DMSO-d_6_): δ (ppm) 1.64–1.75 (m, 1H, CH_2_), 1.76–1.94 (m, 2H, CH_2_), 1.95–2.06 (m, 1H, CH_2_), 2.71–2.83 (m, 1H, CH_2_), 3.74 (d, *J* = 10.4 Hz, 1H, CH), 4.31–4.38 (m, 1H, CH), 6.82 (d, *J* = 8.3 Hz, 1H, ArH), 7.28–7.39 (m, 4H, ArH), 7.46 (s, 1H, ArH), 7.55 (d, *J* = 1.7 Hz, 1H, ArH), 8.50 (s, 1H, NH), 10.47 (s, 1H, NH), 10.57 (s, 1H, NH); ^13^C{1H}-NMR (DMSO-d_6_): δ 27.73, 29.87, 46.88, 56.56, 68.02, 77.02, 80.05, 109.77, 122.27, 123.78, 127.26, 128.77, 129.08, 129.88, 130.52, 133.36, 136.29, 137.46, 142.70, 155.46, 172.43, 175.26; HRMS: calcd. for C_22_H_18_Cl_2_N_4_O_3_^+^ [M+H]^+^: 457.0831, found: 457.0834.

*1-(3-Chloro)phenylhexahydro-1H-pyrrolizine-2-spiro-5'-imidazolidine-2',4'-dione-3-spiro-3''-N-benzylindoline-2''-one* (**4k**). White solid; m.p. 216–218 °C; ^1^H-NMR (DMSO-d_6_): δ (ppm) 1.62–1.75 (m, 1H, CH_2_), 1.76–2.04 (m, 3H, CH_2_), 2.79–2.91 (m, 1H, CH_2_), 3.82 (d, *J* = 10.4 Hz, 1H, CH), 4.40–4.50 (m, 1H, CH), 4.72 (d, *J* = 15.9 Hz, 1H, CH_2_), 5.05 (d, *J* = 15.9 Hz, 1H, CH_2_), 6.83 (d, *J* = 7.8 Hz, 1H, ArH), 7.04–7.13 (m, 1H, ArH), 7.12–7.42 (m, 9H, ArH), 7.50 (s, 1H, ArH), 7.62 (d, *J* = 7.5 Hz, 1H, ArH), 8.55 (s, 1H, NH), 10.46(s, 1H, NH); ^13^C{1H}-NMR (DMSO-d_6_): δ 27.68, 29.84, 43.13, 46.86, 56.63, 68.00, 77.00, 80.02, 109.74, 122.25, 123.76, 127.23, 127.78, 128.03, 128.67, 128.82, 129.06, 129.78, 129.92, 130.49, 133.35, 136.27, 137.45, 142.69, 155.43, 172.43, 175.24; HRMS: calcd. for C_29_H_25_ClN_4_O_3_^+^ [M+H]^+^: 513.1693, found: 513.1693.

*1-(4-Trifluoromethy)phenylhexahydro-1H-pyrrolizine-2-spiro-5'-imidazolidine-2',4'-dione-3-spiro-3''-indoline-2''-one* (**4l**). White solid; m.p. 239–241 °C; ^1^H-NMR (DMSO-d_6_): δ 1.60–1.72 (m, 1H, CH_2_), 1.73–1.87 (m, 1H, CH_2_), 1.87–2.06 (m, 2H, CH_2_), 2.87–2.98 (m, 1H, CH_2_), 3.82 (d, *J* = 10.3 Hz, 1H, CH), 4.41–4.51 (m, 1H, CH), 6.81 (d, *J* = 7.7 Hz, 1H, ArH), 6.98–7.06 (m, 1H, ArH), 7.21–7.30 (m, 1H, ArH), 7.52 (d, *J* = 7.6 Hz, 1H, ArH), 7.60 (d, *J* = 8.2 Hz, 2H, ArH), 7.69 (d, *J* = 8.2 Hz, 2H, ArH), 8.30 (s, 1H, NH), 10.45 (s, 1H, NH), 10.48 (s, 1H, NH); ^13^C{1H}-NMR (DMSO-d_6_): δ 27.02, 29.38, 46.55, 56.55, 67.65, 76.82, 79.50, 109.87, 121.08, 122.92, 124.05, 125.06, 125.62, 127.86, 128.18, 128.35, 129.50, 129.81, 130.59, 139.88, 142.27, 154.97, 172.52, 176.28; HRMS: calcd. for C_23_H_19_F_3_N_4_O_3_^+^ [M+H]^+^: 457.1486, found: 457.1488.

*1-(3-Methoxy)phenylhexahydro-1H-pyrrolizine-2-spiro-5'-imidazolidine-2',4'-dione-3-spiro-3''-indoline-2''-one* (**4m**). Yellow solid; m.p. 177–179 °C; ^1^H-NMR (400 MHz, DMSO-d6): δ (ppm) 1.57–1.70 (m, 1H, CH_2_), 1.72–1.87 (m, 1H, CH_2_), 1.87–2.04 (m, 2H, CH_2_), 2.83–2.95 (m, 1H, CH_2_), 3.67 (d, *J* = 10.3 Hz, 1H, CH), 4.35–4.46 (m, 1H, CH), 6,77–6.86 (m, 2H, ArH), 6.89 (d, *J* = 7.6 Hz, 1H, ArH), 6.97–7.06 (m, 2H, ArH), 7.16–7.28 (m, 2H, ArH), 7.49 (d, *J* = 7.5 Hz, 1H, ArH), 8.07 (s, 1H, NH), 10.34 (s, 1H, NH), 10.40 (s, 1H, NH); ^13^C{1H}-NMR (100 MHz, DMSO-d6): δ 27.16, 29.63, 46.50, 54.93, 56.85, 59.75, 67.50, 76.83, 79.45, 109.82, 112.82, 115.22, 121.01, 121.95, 124.25, 128.34, 129.25, 136.42, 142.21, 155.00, 159.07, 172.55, 176.43; HRMS: calcd. for C_23_H_22_N_4_O_4_^+^ [M+H]+: 419.1718, found: 419.1719.

*7-Phenyl-5-spiro-3'-indoline-2'-one-hexahydropyrrolo[1,2-c]thiazole-6-spiro-5''-imidazolidine-2'',4''-dione* (**4n**). White solid; m.p. 200–202 °C; ^1^H-NMR (DMSO-d6): δ (ppm) 2.87 (dd, *J* = 11.2, 4.1 Hz, 1H, CH_2_). 3.07 (dd, *J* = 11.1, 6.8 Hz, 1H, CH_2_), 3.54 (d, *J* = 9.4 Hz, 1H, CH), 3.71–3.80 (m, 2H, CH_2_), 4.63 (m, 1H, CH), 6.79 (d, *J* = 7.7 Hz, 1H, ArH), 7.01 (m, 1H, ArH), 7.21–7.35 (m, 4H, ArH), 7.40 (d, *J* = 6.6 Hz, 1H, ArH), 7.73 (d, *J* = 7.6 Hz, 1H, ArH), 8.58 (s, 1H, NH), 10.30 (s, 1H, NH), 10.48 (s, 1H, NH); ^13^C{1H}-NMR (DMSO-d_6_): δ 34.80, 51.90, 55.13, 70.18, 77.11, 78.29, 109.70, 120.70, 122.78, 127.84, 128.31, 129.21, 129.67, 129.83, 133.78, 141.79, 154.84, 170.67, 175.45; HRMS: calcd. for C_21_H_18_N_4_O_3_S^+^ [M+H]^+^: 407.1176, found: 407.1178.

### 3.3. Crystallographic Data and Molecular Structure of Compound **4b** [32]

C_29_H_26_N_4_O_3_•C_2_H_6_O, M = 524.61, triclinic, a = 9.8656(7) Å, b = 11.0707(8) Å, c = 13.5643(10) Å, α = 98.6770(10)°, β = 110.6190(10)°, γ = 103.4270(10)°, V = 1303.90(16) Å3, T = 100(2) K, space group P1, Z = 2, μ(MoKα) = 0.090 mm-1, 18612 reflections measured, 7266 independent reflections (Rint = 0.0225). The final R1 values were 0.0436 (I > 2σ(I)). The final wR(F2) values were 0.1160 (I > 2σ(I)). The final R1 values were 0.0560 (all data). The final wR(F2) values were 0.1250 (all data). The goodness of fit on F2 was 1.021.

## 4. Conclusions

In conclusion, we have successfully developed an efficient method for the synthesis of potentially biologically active a series of novel dispirocycloadducts via a three-component 1,3-dipolar cycloaddition reaction of azomethine ylides. This method has the advantages of convenient operation, the availability of starting materials, mild reaction conditions employed, high yields and high efficiency, as well as the complete regio- and stereoselectivity observed. Further studies to acquire more information about the pharmacological activity of these compounds are in progress in our laboratory.

## Supplementary Materials

Supplementary materials can be accessed at: http://www.mdpi.com/1420-3049/18/5/5142/s1.
