# Supplementary material for: A Facile Synthesis of Functionalized Dispirooxindole Derivatives via a Three-Component 1,3-Dipolar Cycloaddition Reaction
Source: Molecules. 2013 May 3;18(5):5142–54. doi: 10.3390/molecules18055142 (PMC6270352; doi:10.3390/molecules18055142)

Supporting Information

^{1}H, ^{13}C-NMR and HRMS Spectras

Figure S1. ^{1}H-NMR of Compound **4a**.

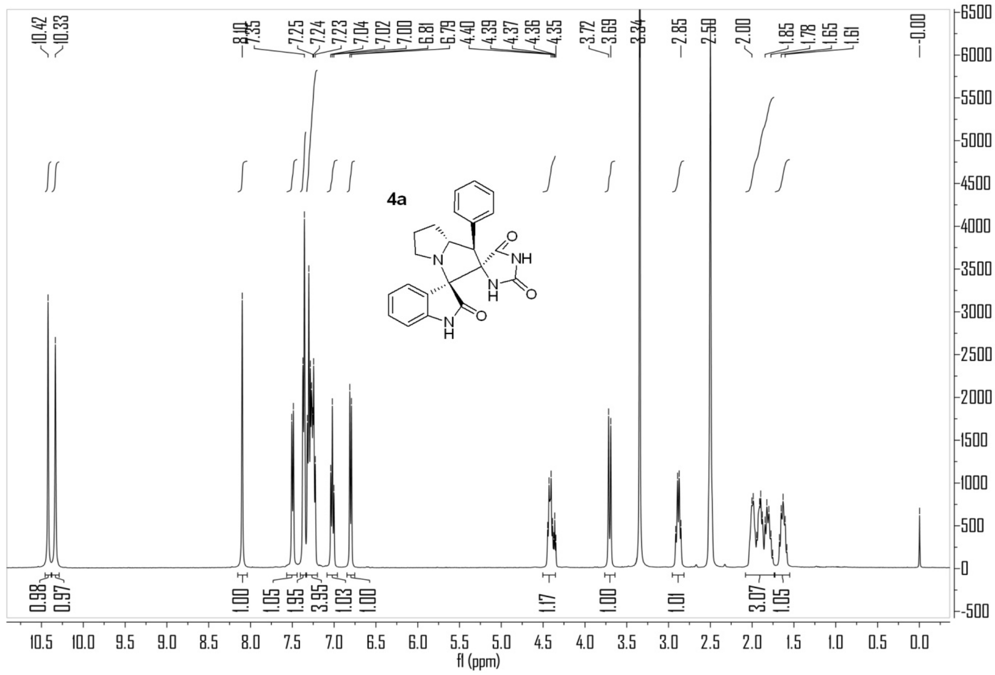

Figure S2. ^{13}C-NMR of Compound **4a**.

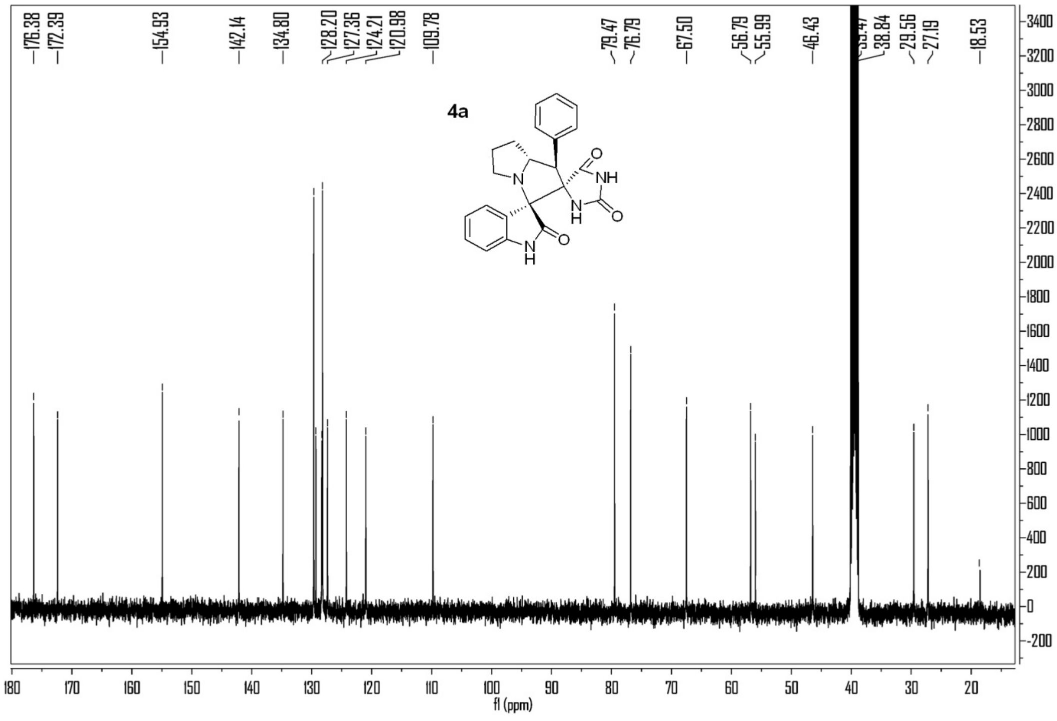

Figure S3. HRMS of Compound **4a**.

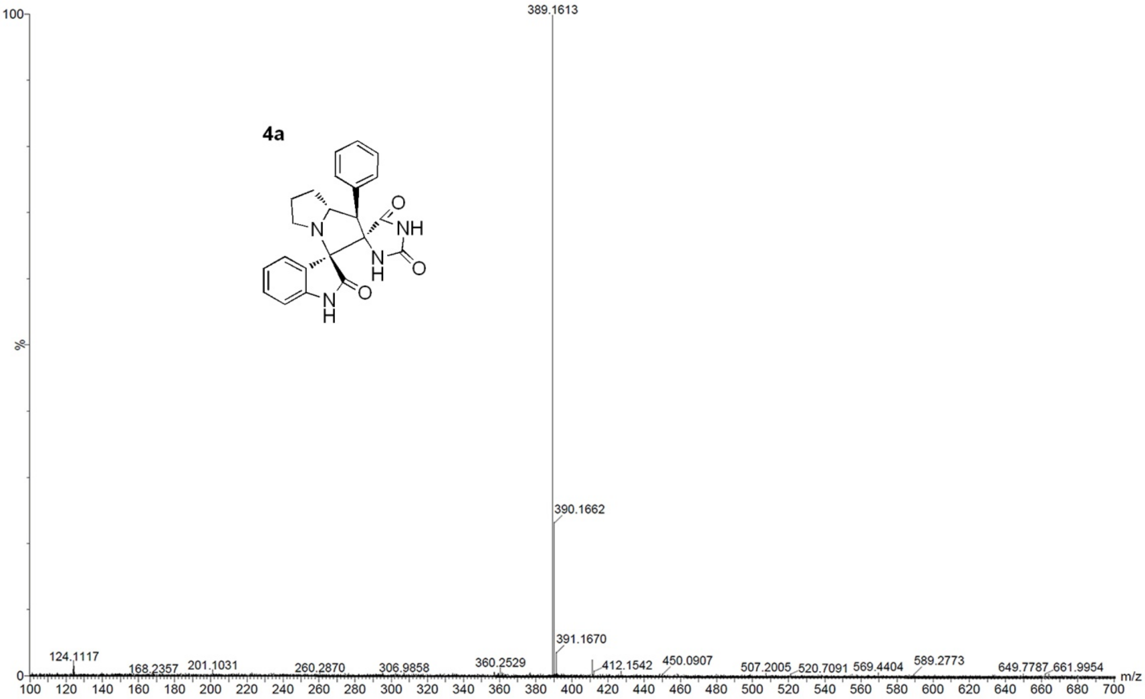

Figure S4. ^{1}H-NMR of Compound **4b**.

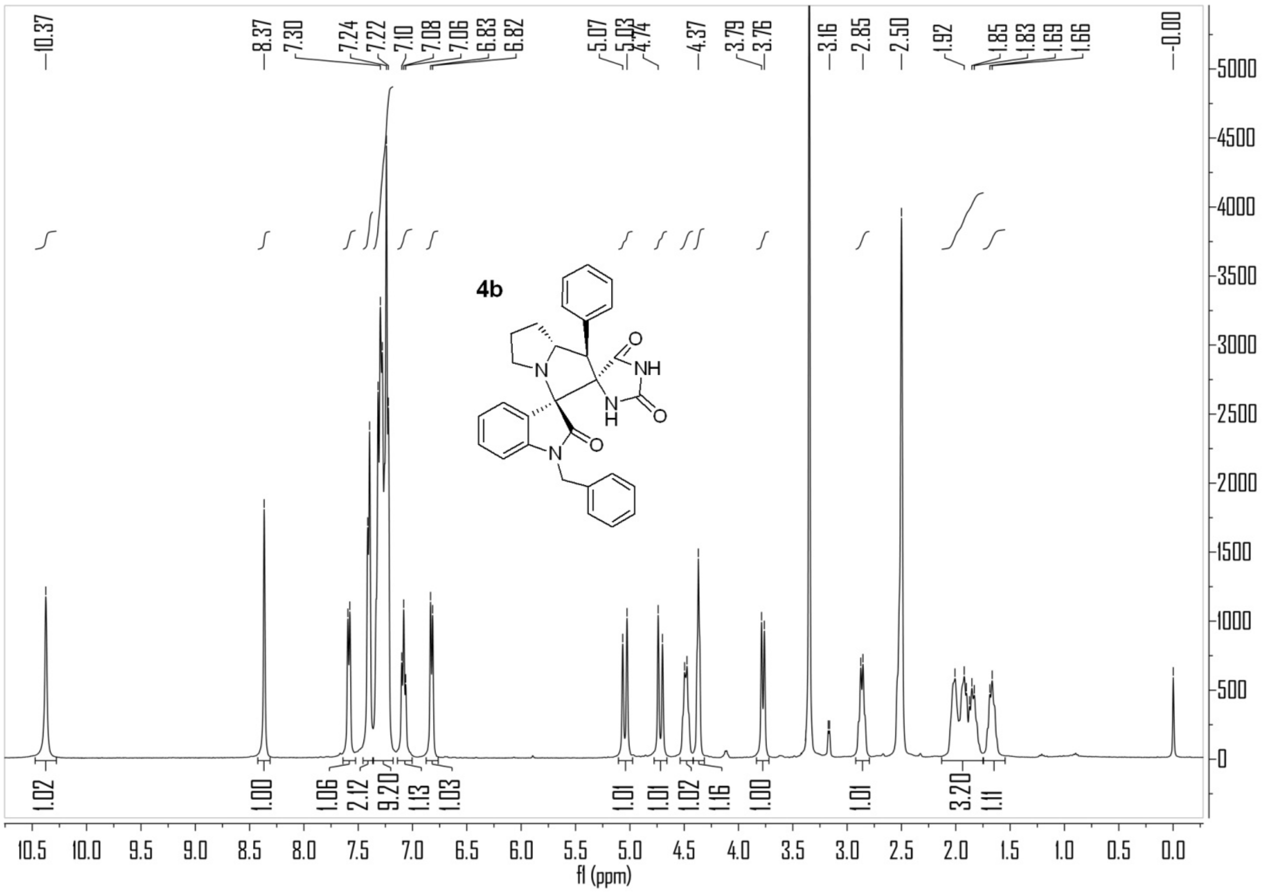

Figure S5. ^{13}C-NMR of Compound **4b**.

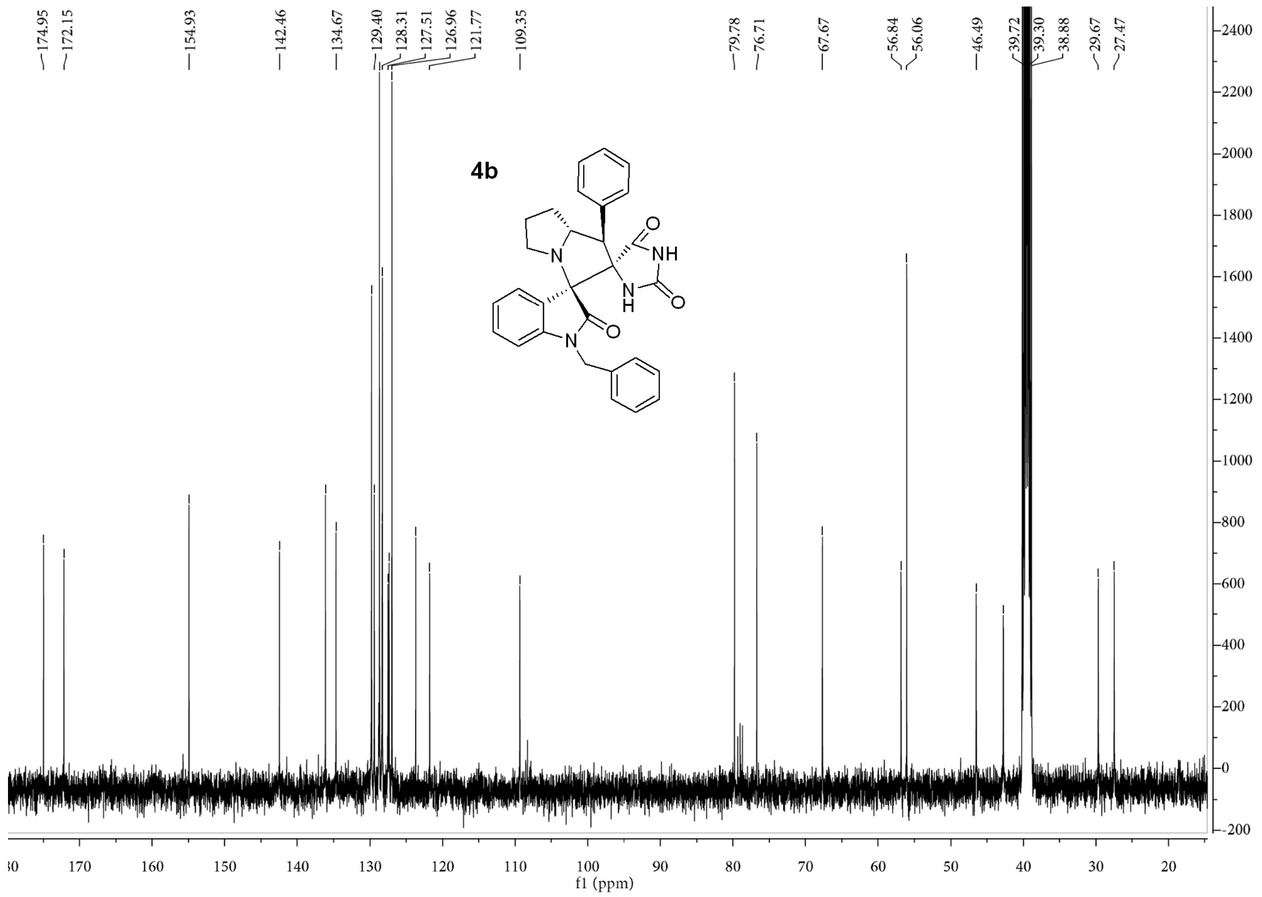

Figure S6. HRMS of Compound **4b**.

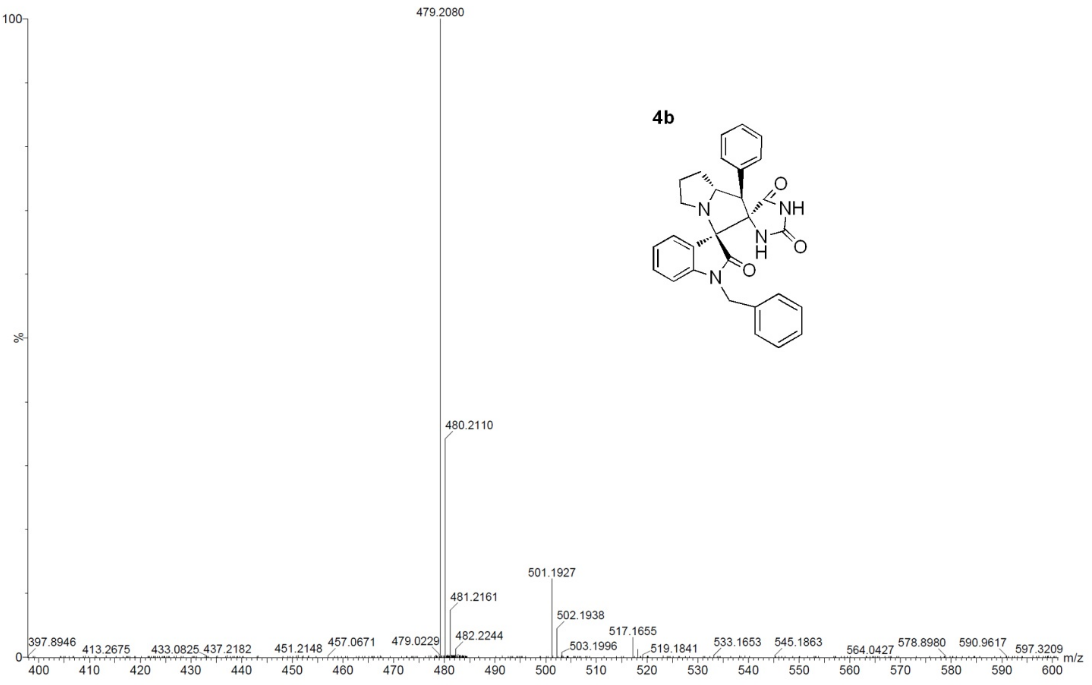

Figure S7. ^{1}H-NMR of Compound **4c**.

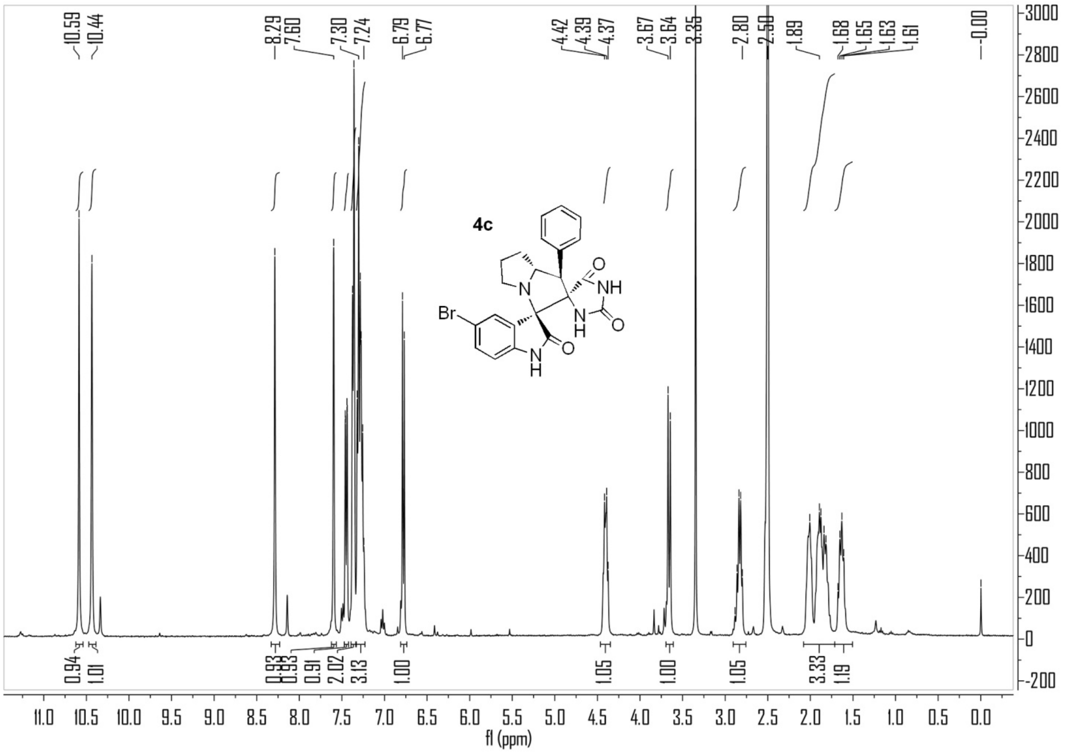

Figure S8. ^{13}C-NMR of Compound **4c**.

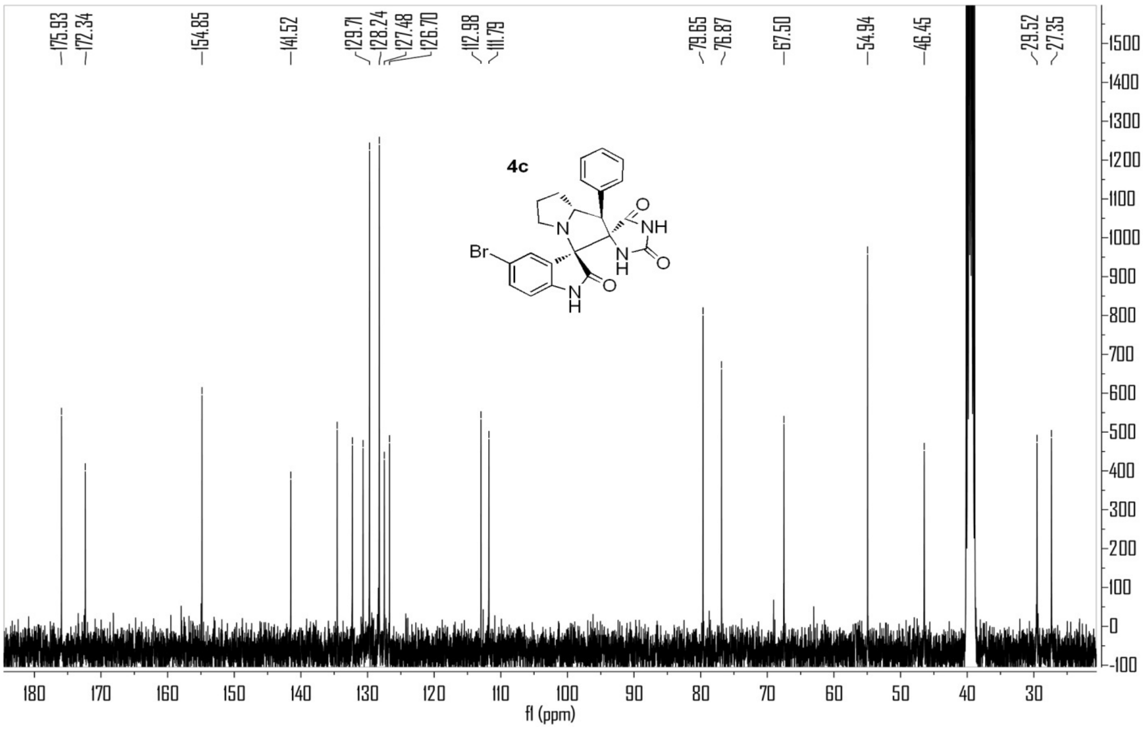

Figure S9. HRMS of Compound **4c**.

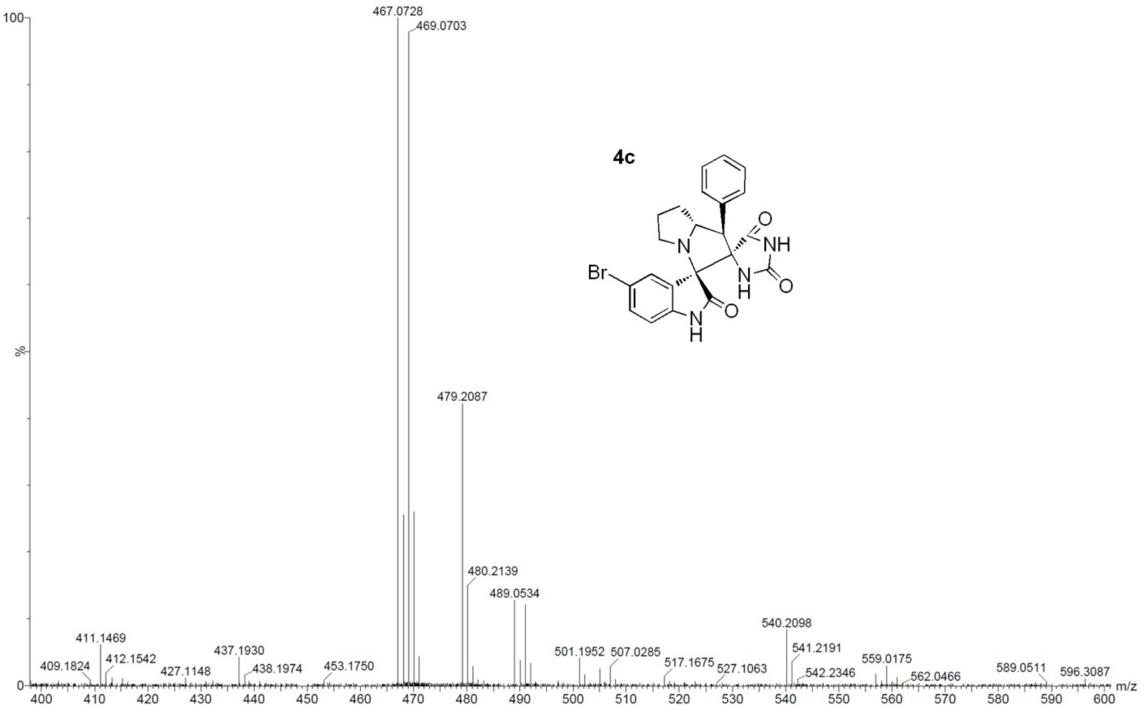

Figure S10. ^{1}H-NMR of Compound **4d**.

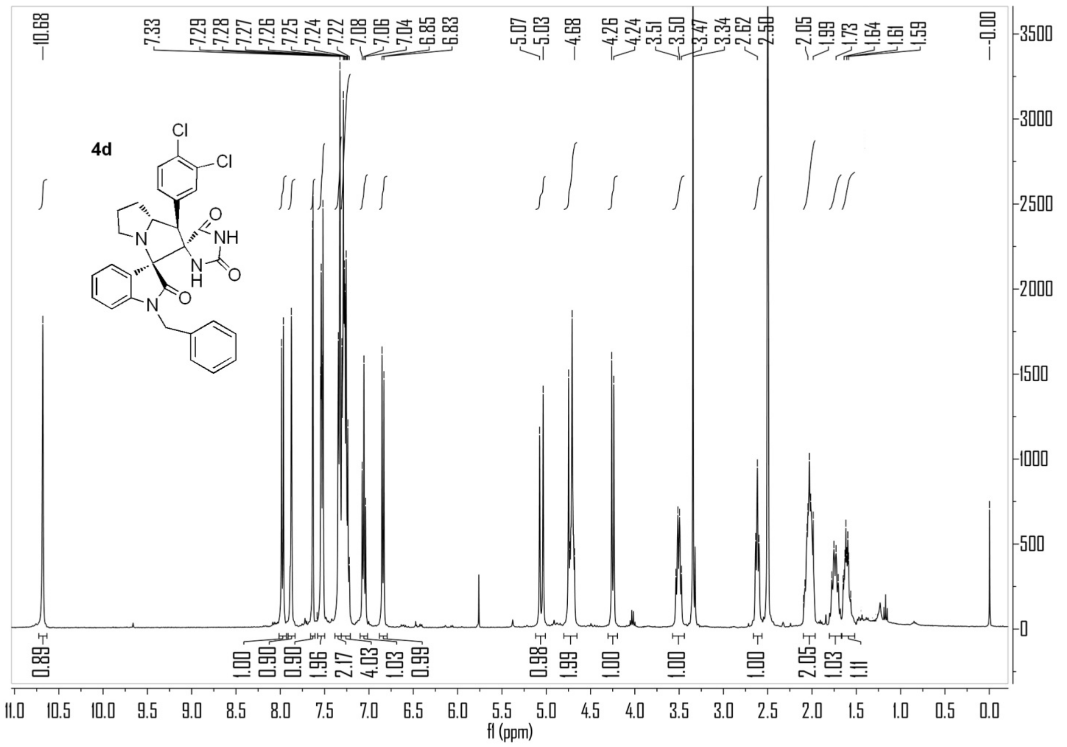

Figure S11. ^{13}C-NMR of Compound **4d**.

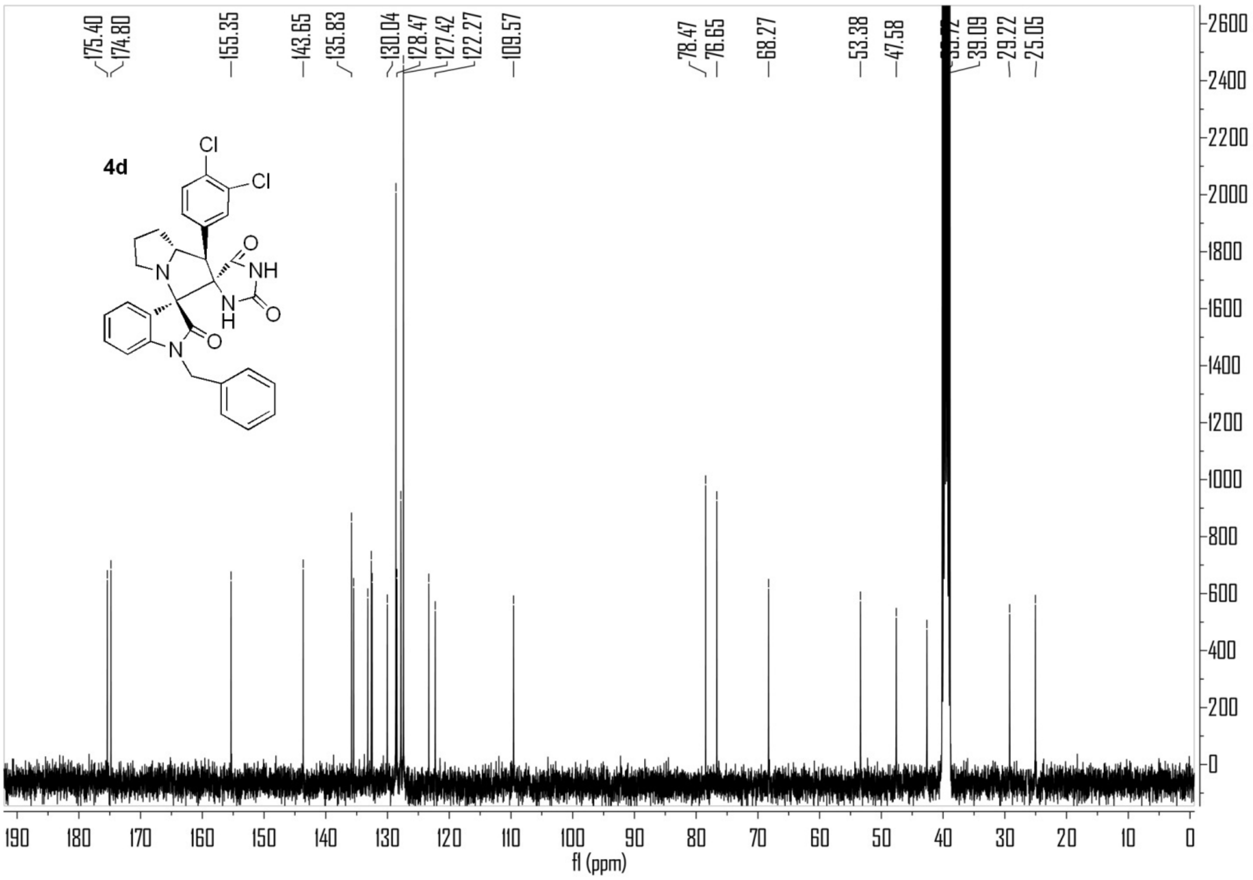

Figure S12. HRMS of Compound **4d**.

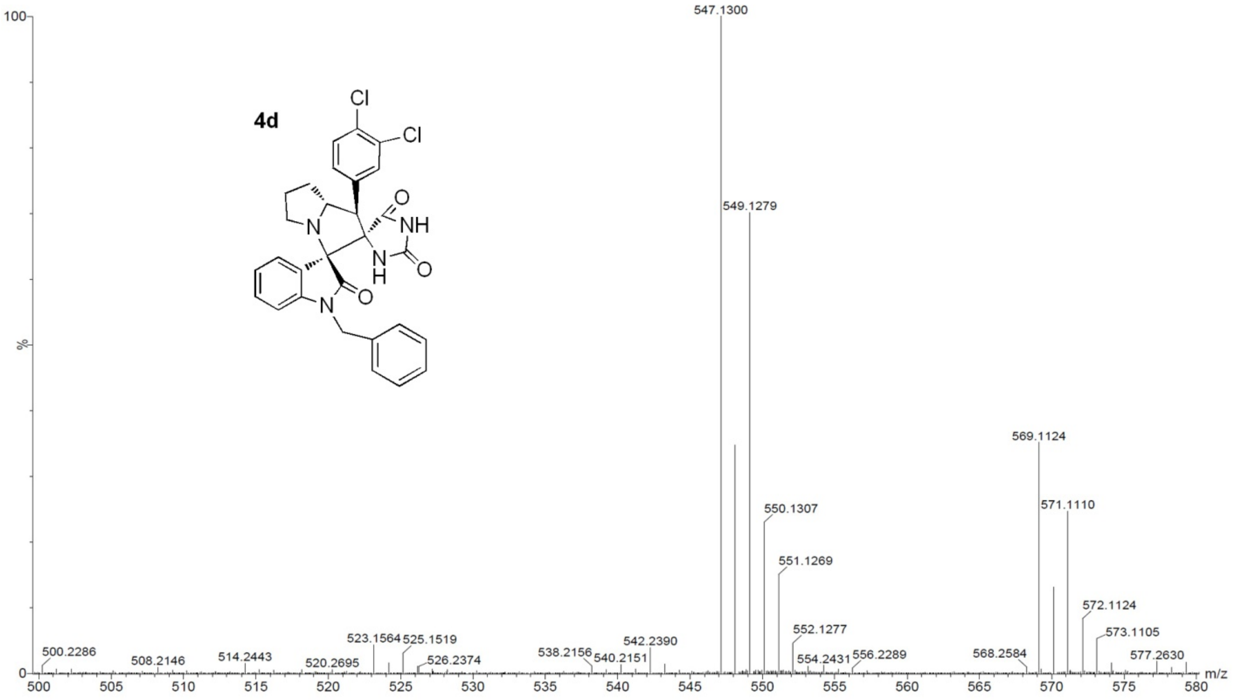

Figure S13. ^{1}H-NMR of Compound **4e**.

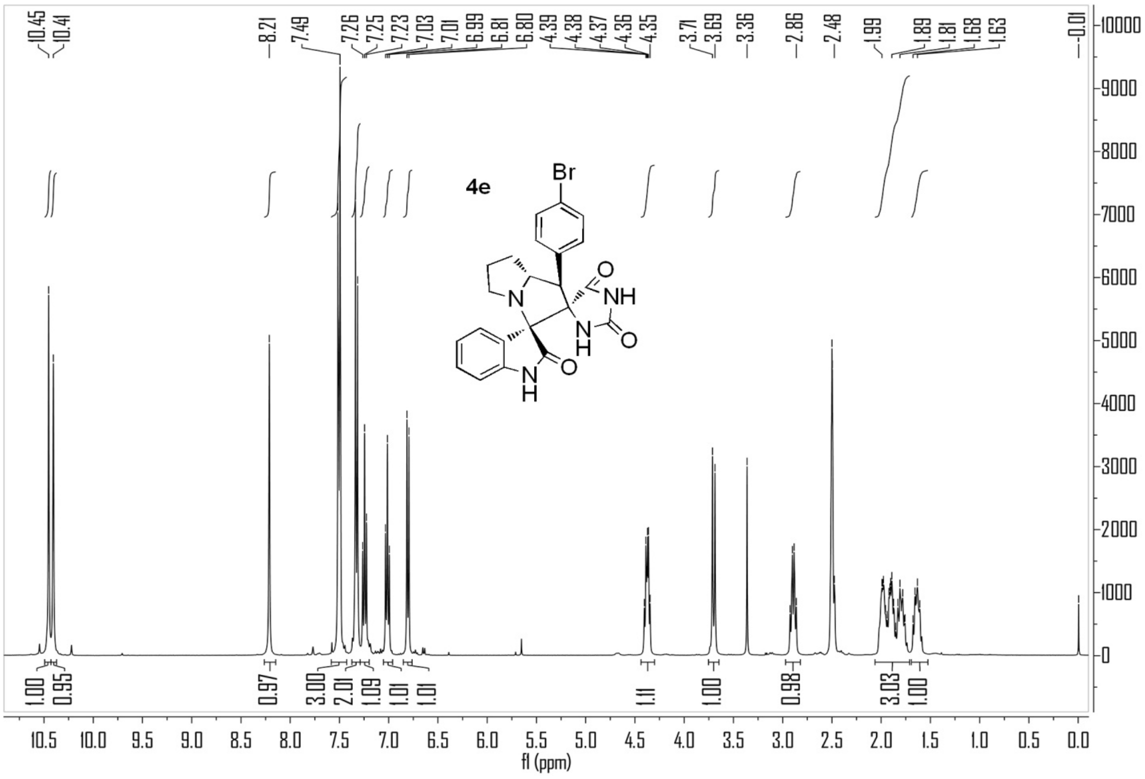

Figure S14. ^{13}C-NMR of Compound **4e**.

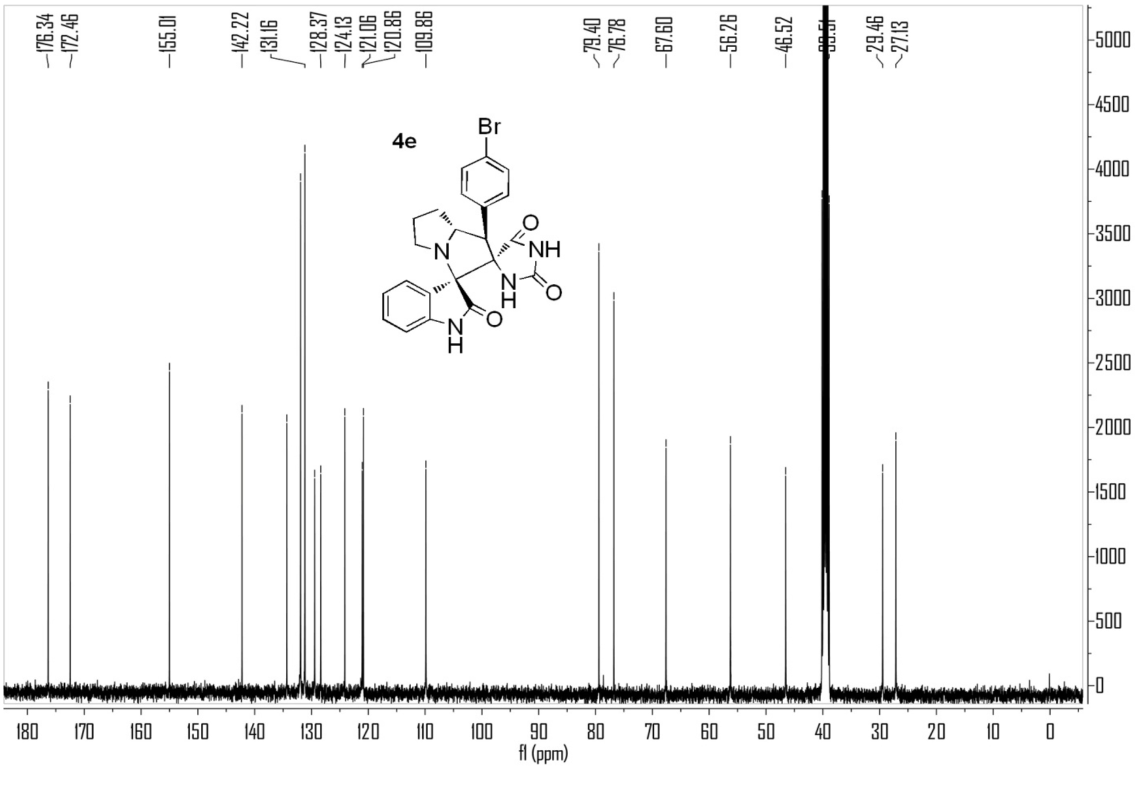

Figure S15. HRMS of Compound **4e**.

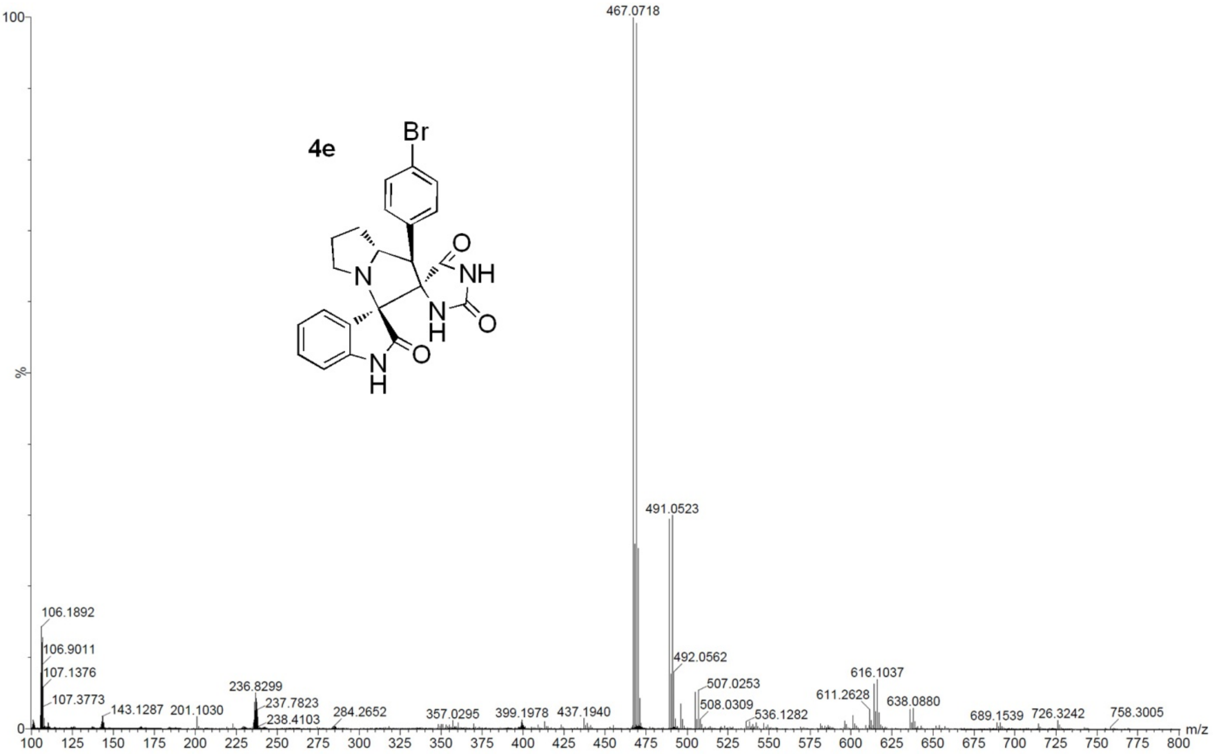

Figure S16. ^{1}H-NMR of Compound **4f**.

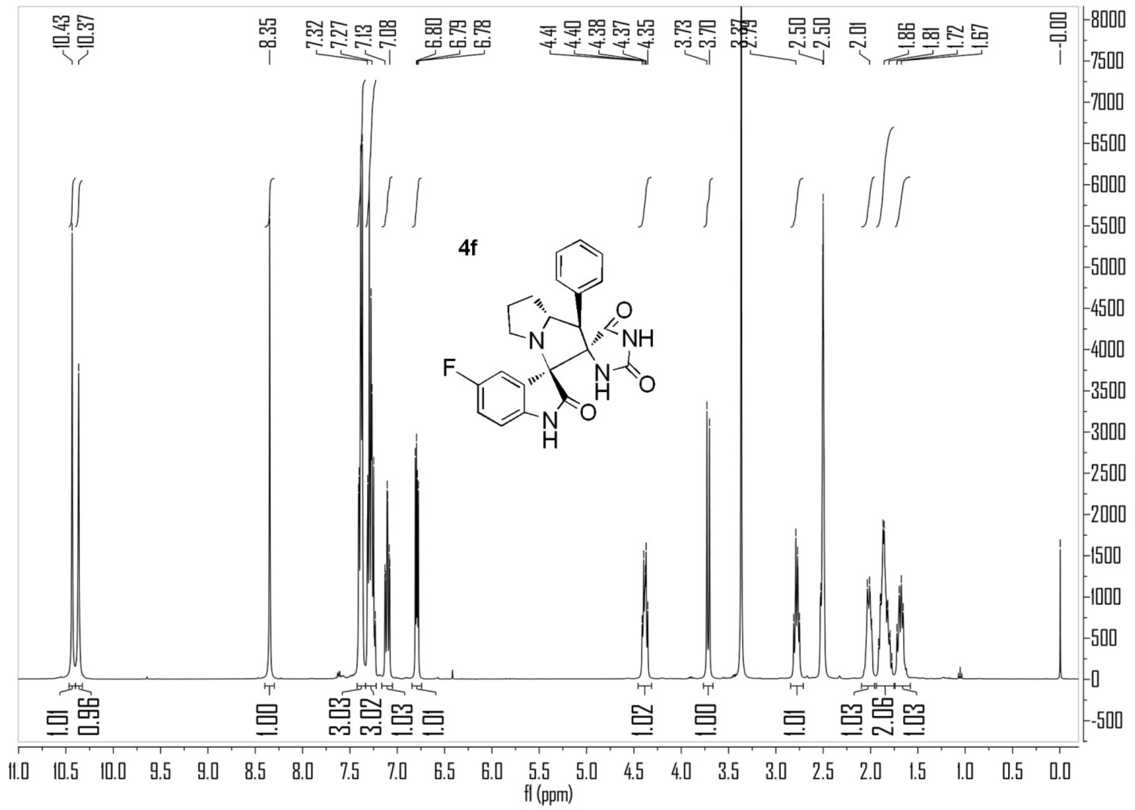

Figure S17. ^{13}C-NMR of Compound **4f**.

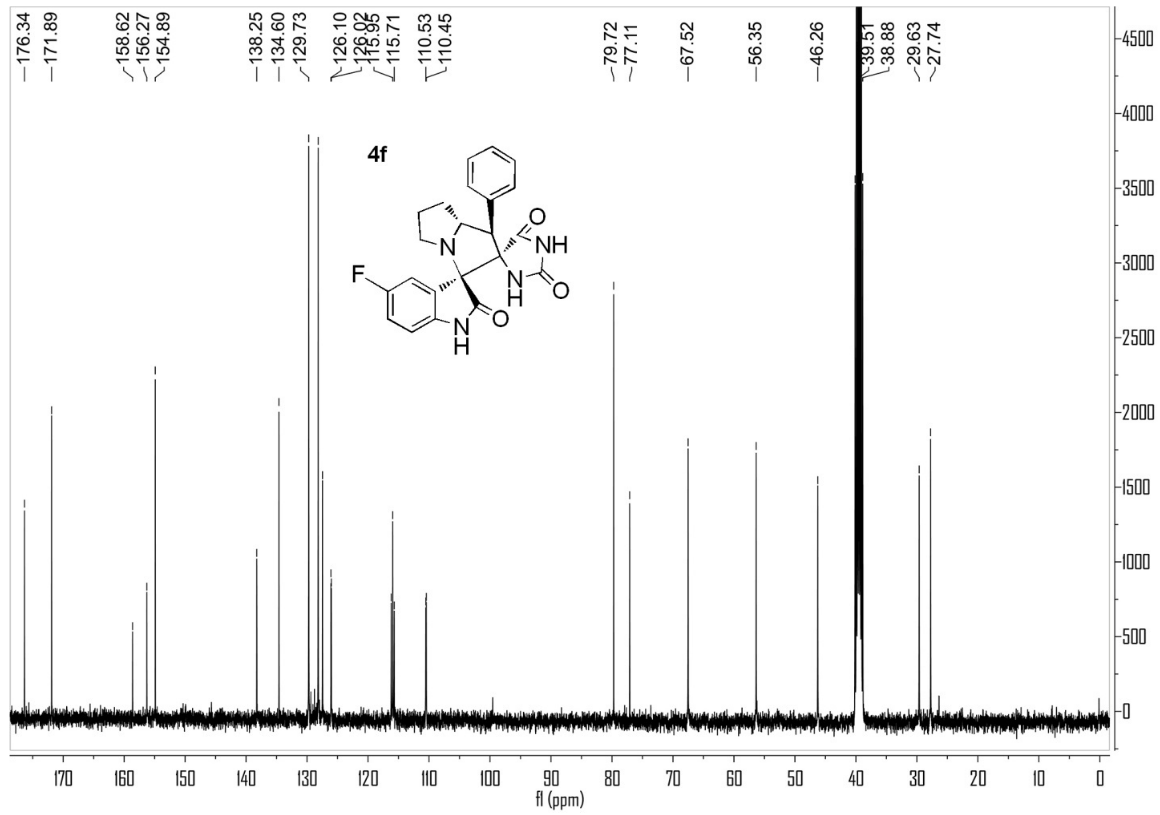

Figure S18. HRMS of Compound **4f**.

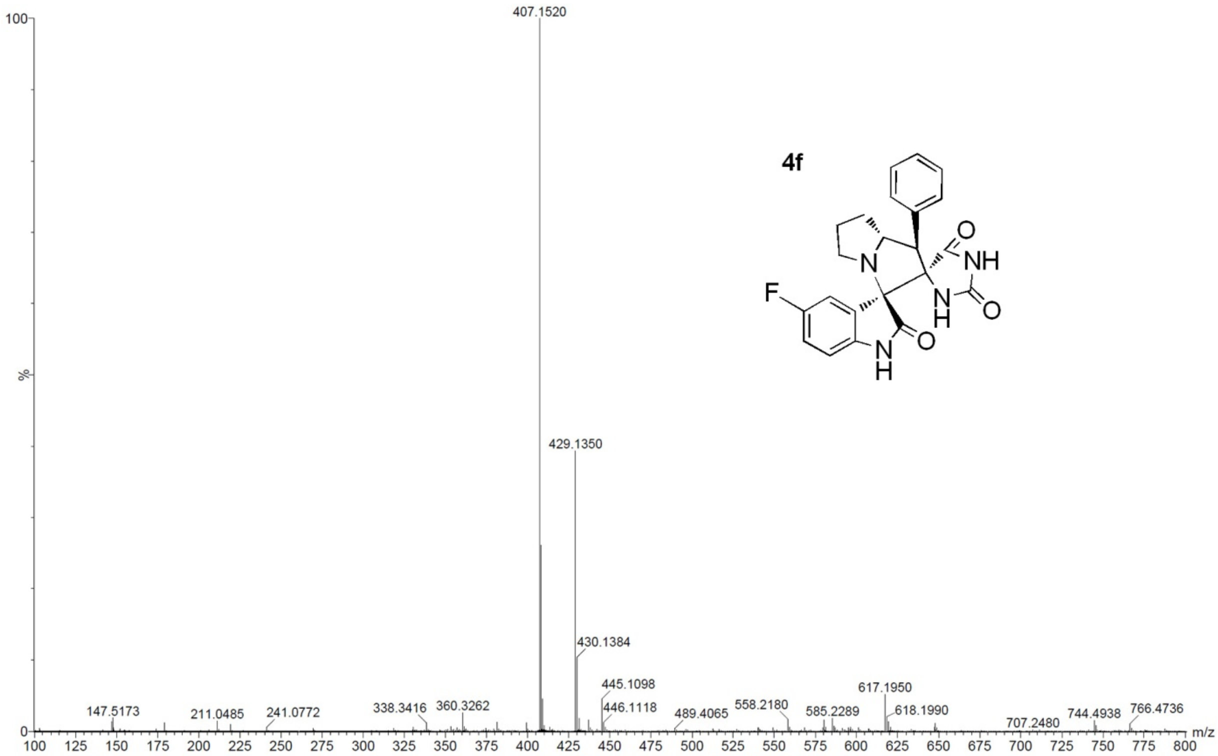

Figure S19. ^{1}H-NMR of Compound **4g**.

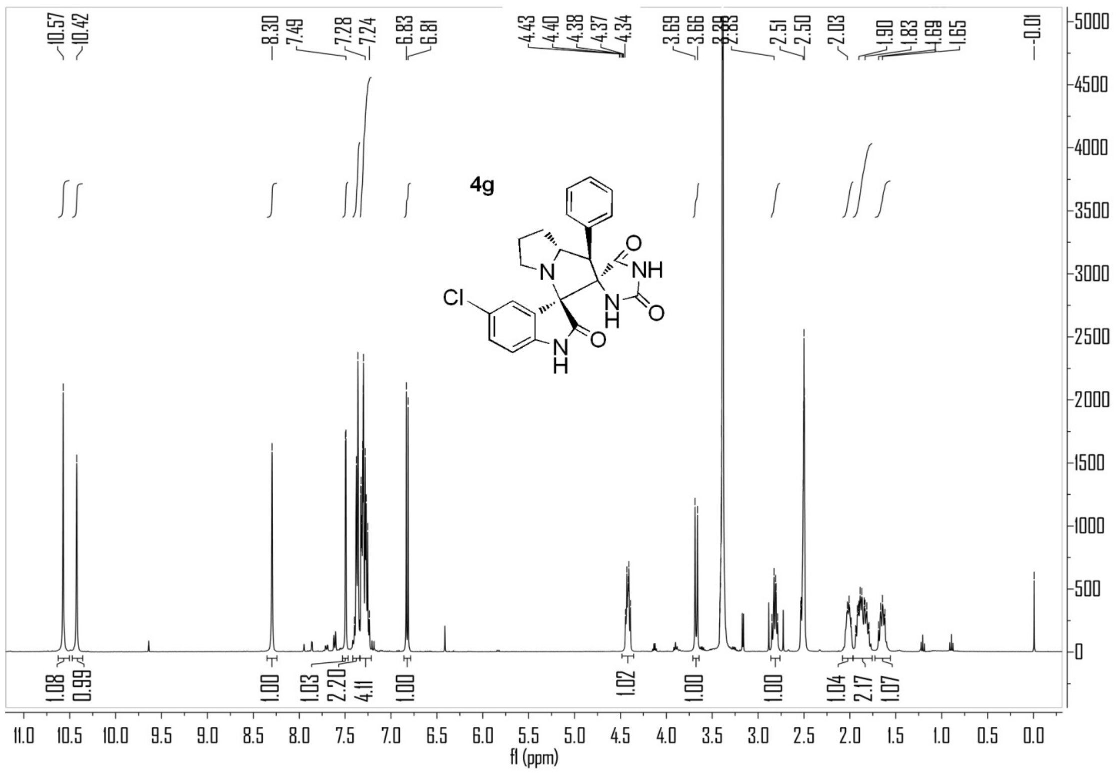

Figure S20. ^{13}C-NMR of Compound **4g**.

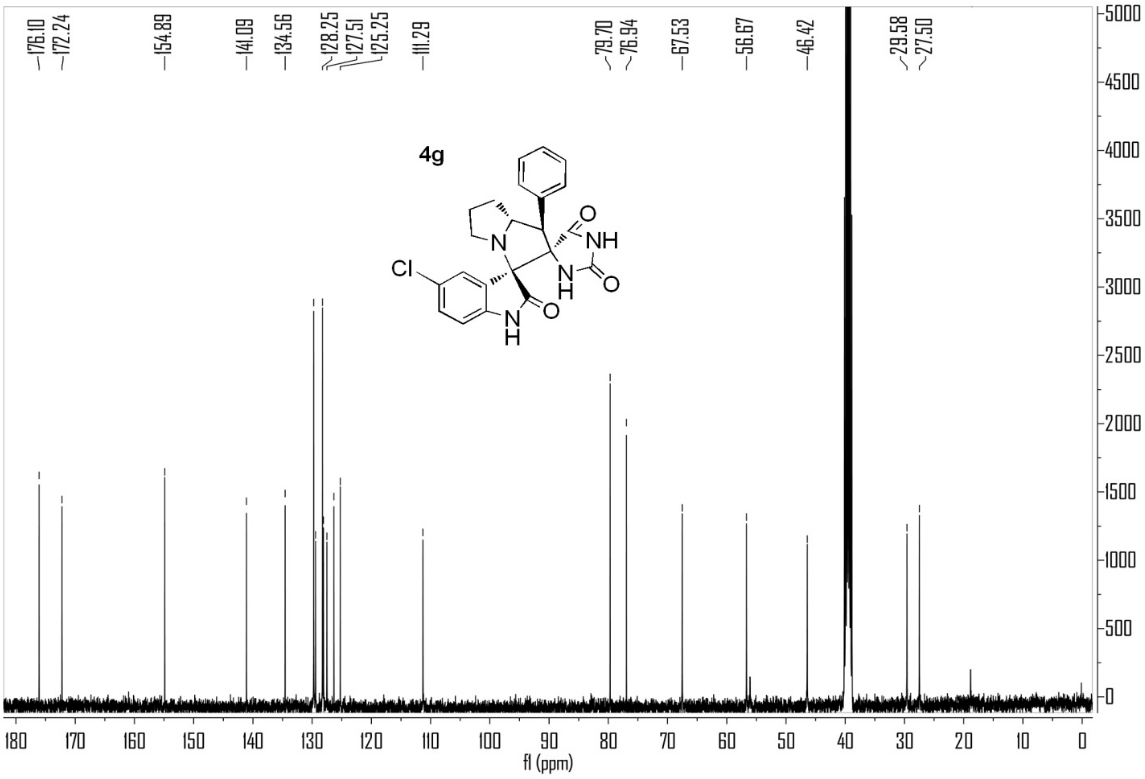

Figure S21. HRMS of Compound **4g**.

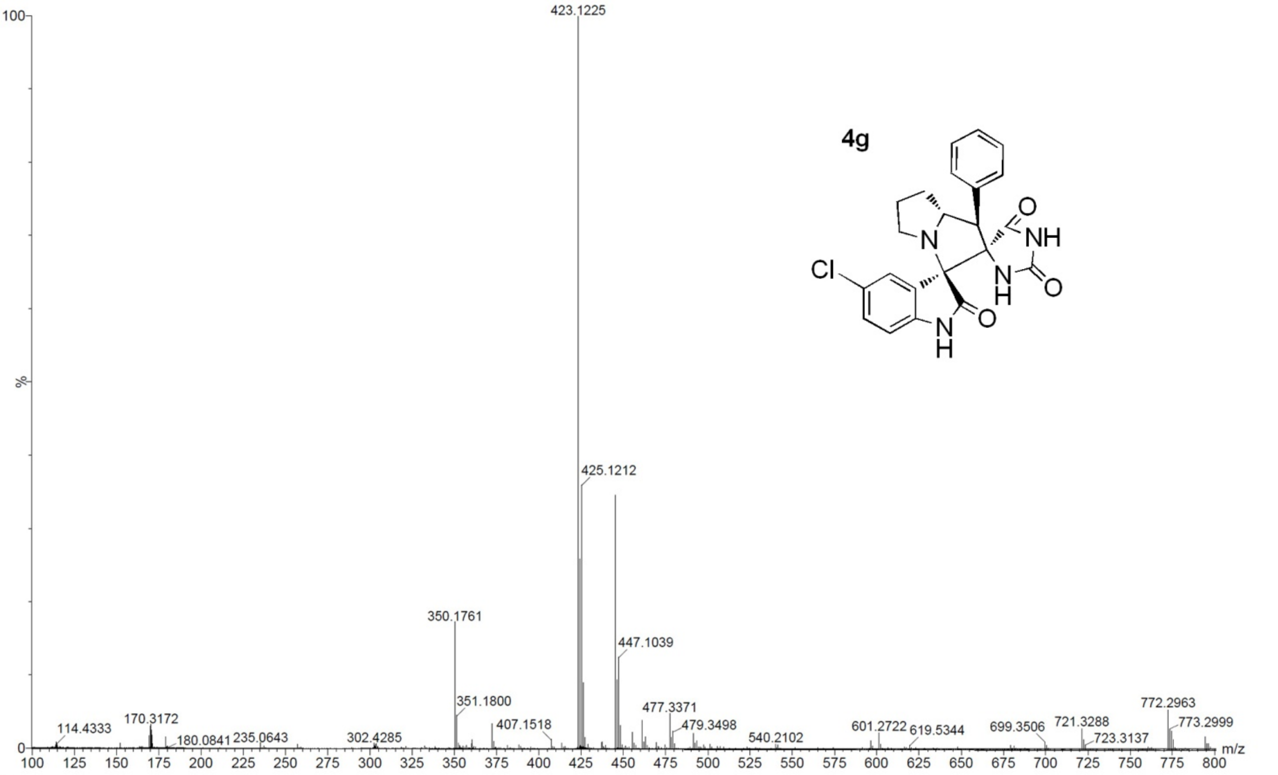

Figure S22. ^{1}H-NMR of Compound **4h**.

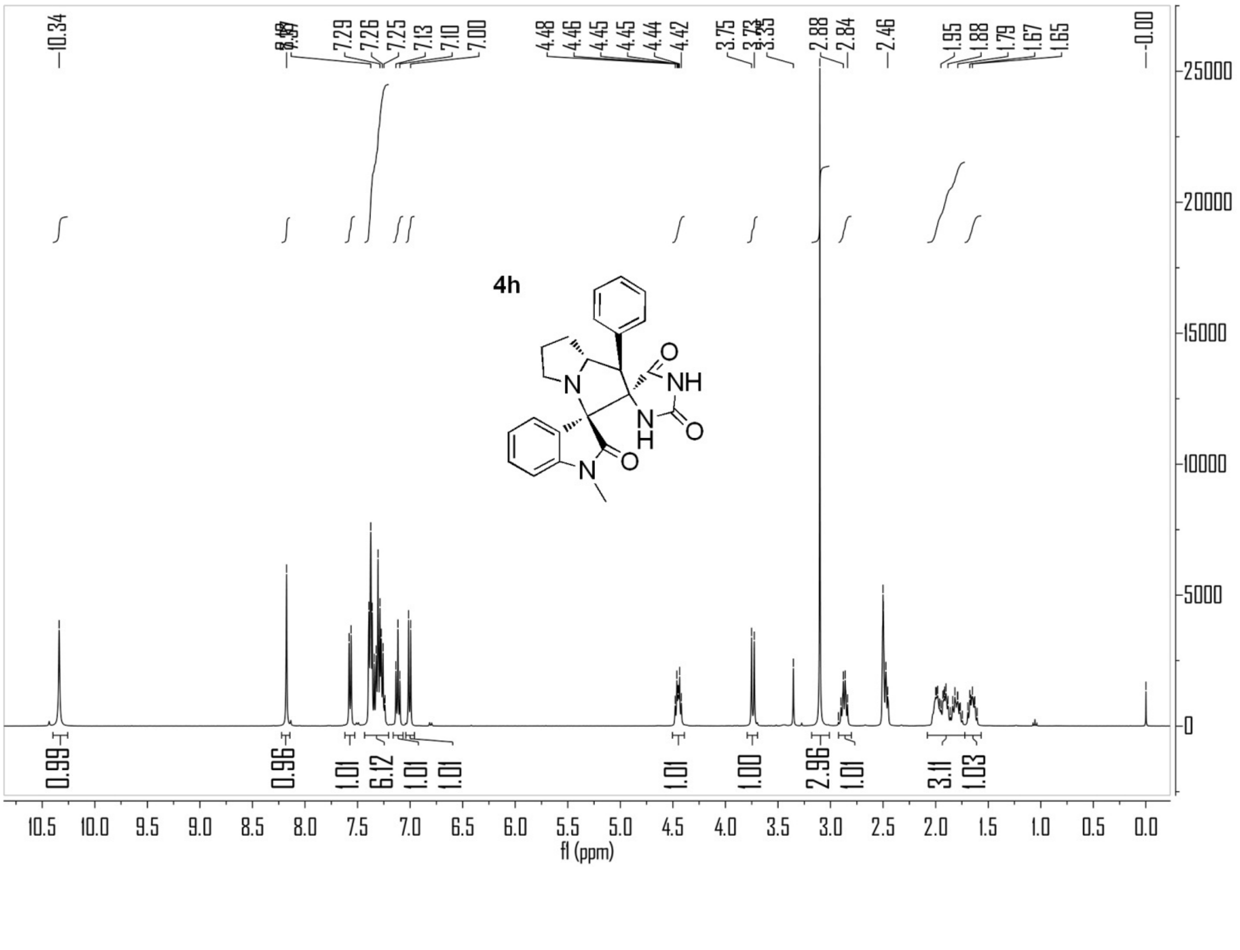

Figure S23. ^{13}C-NMR of Compound **4h**.

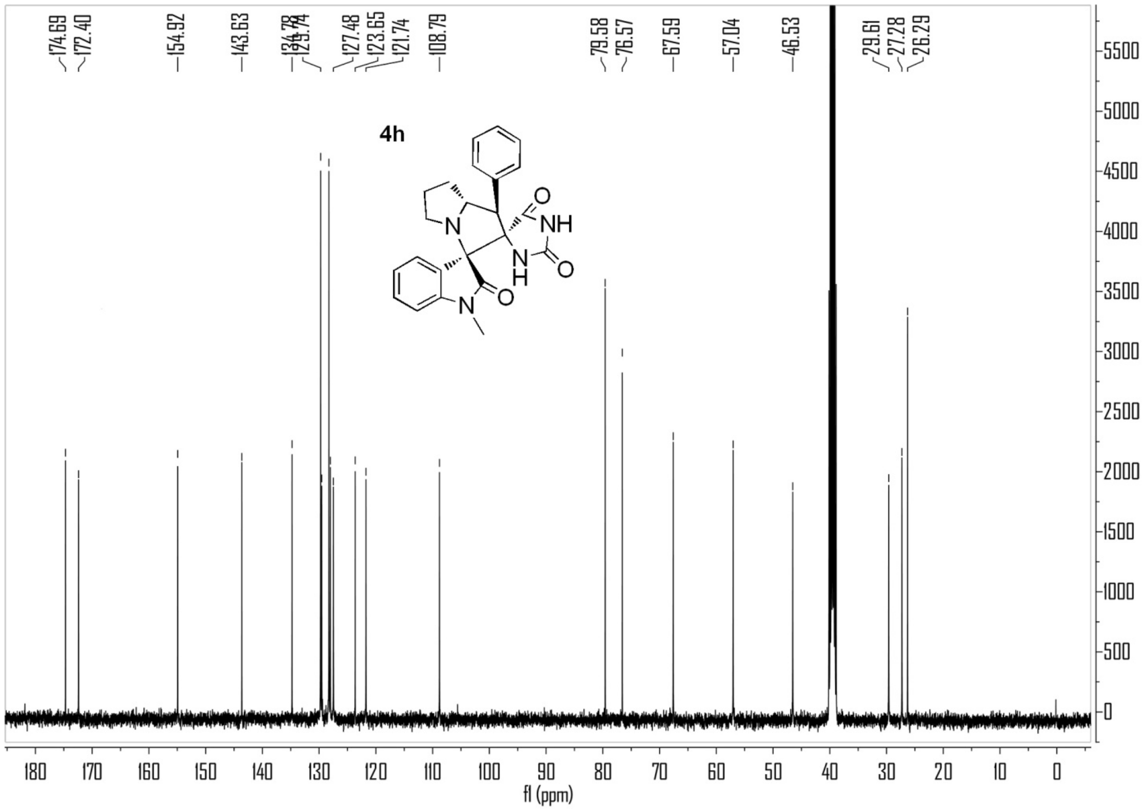

Figure S24. HRMS of Compound **4h**.

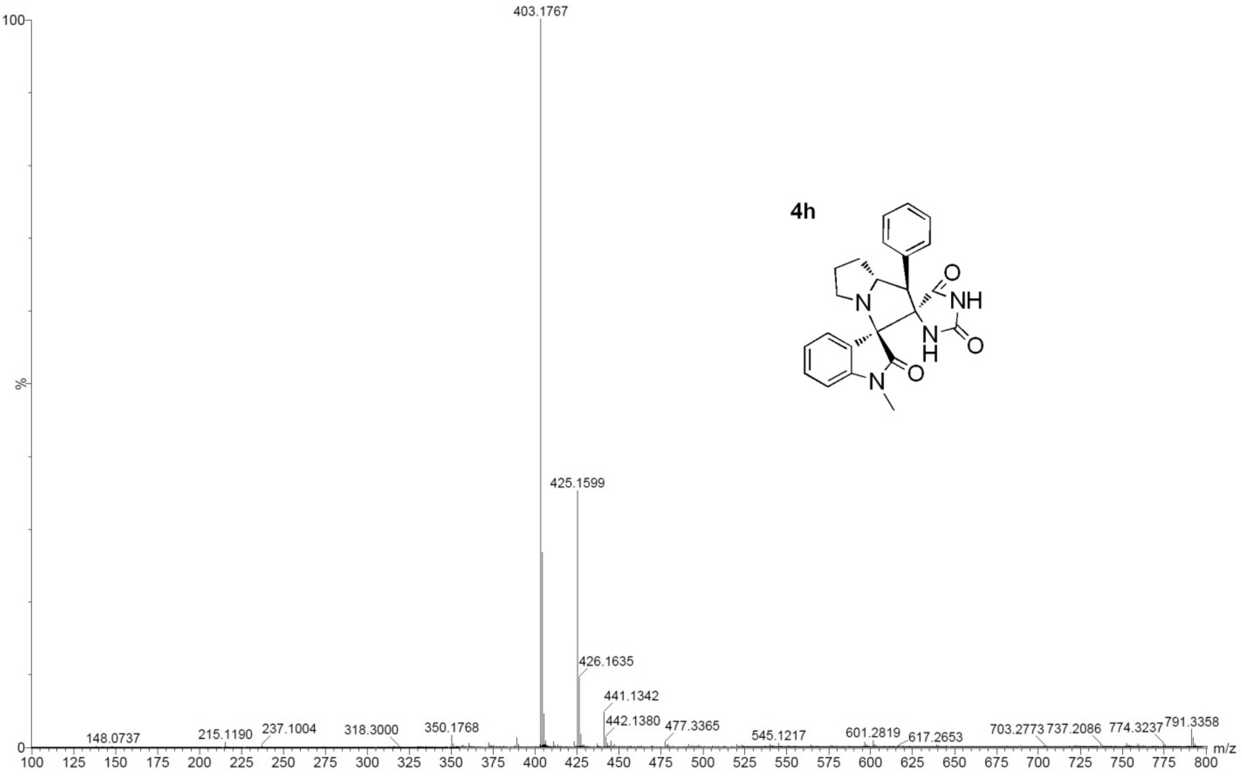

Figure S25. ^{1}H-NMR of Compound **4i**.

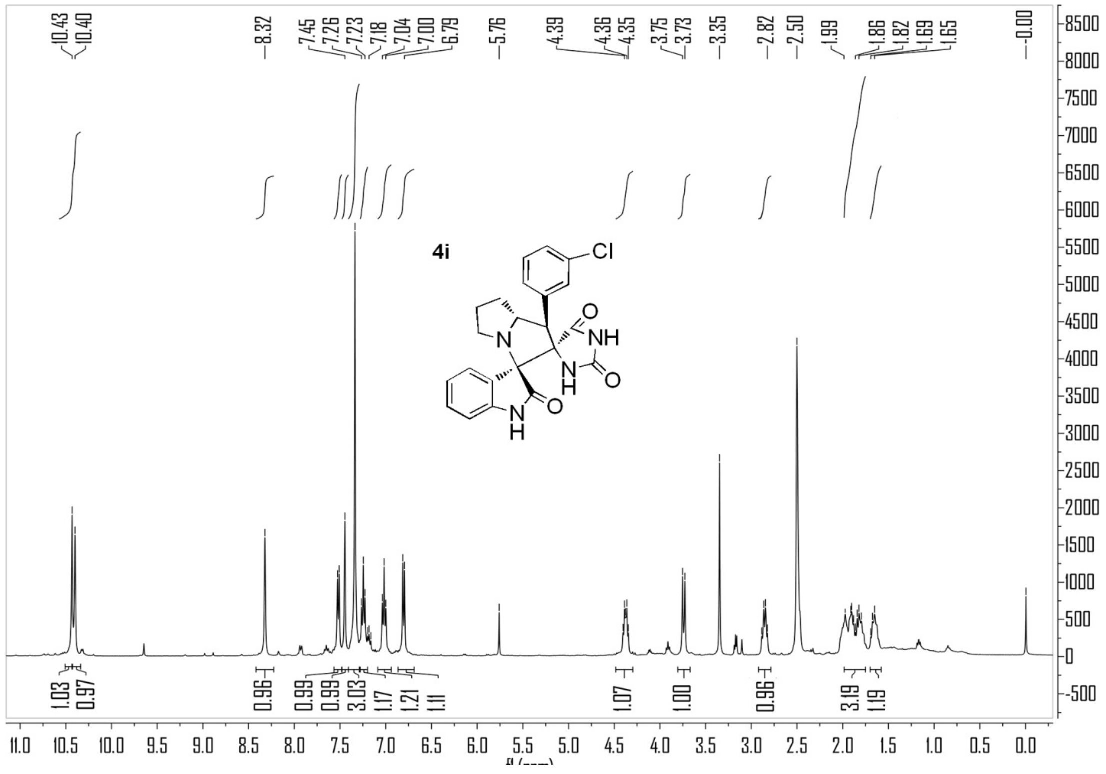

Figure S26. ^{13}C-NMR of Compound **4h**.

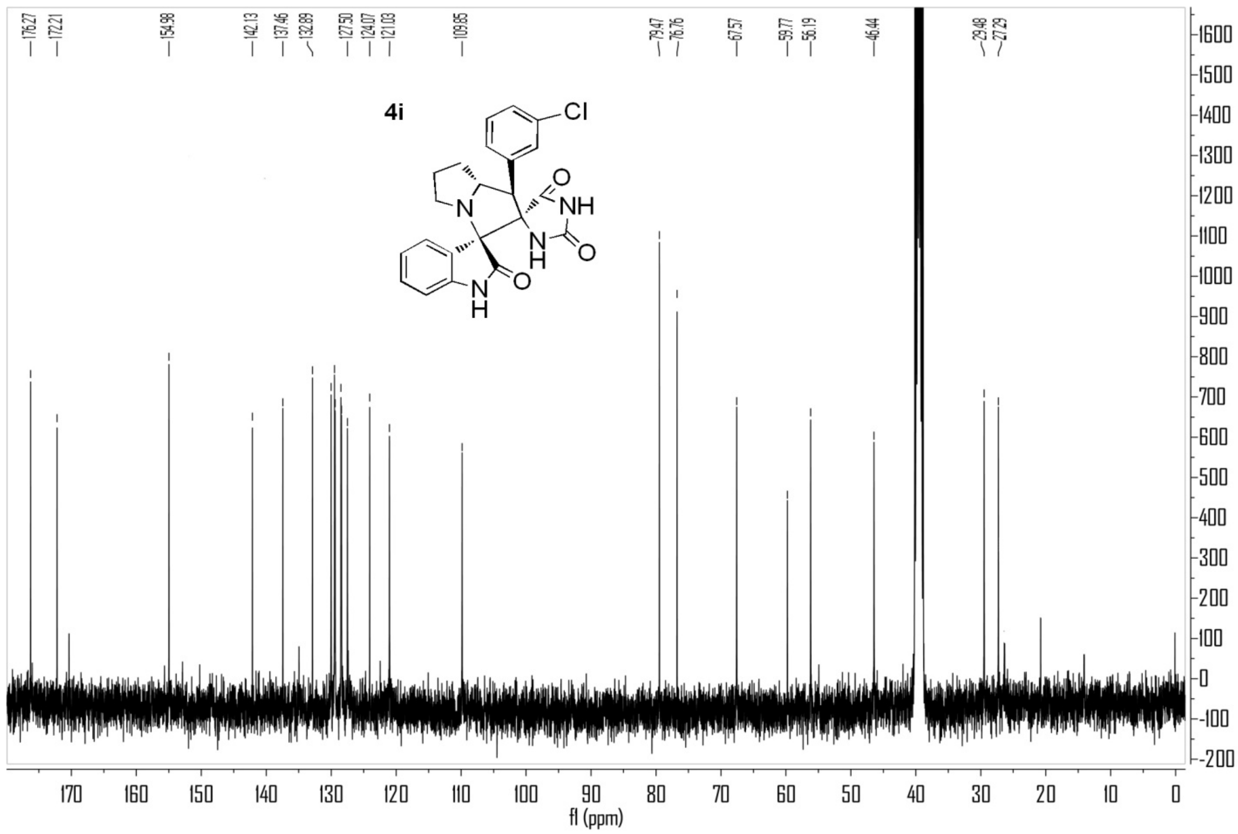

Figure S27. HRMS of Compound **4i**.

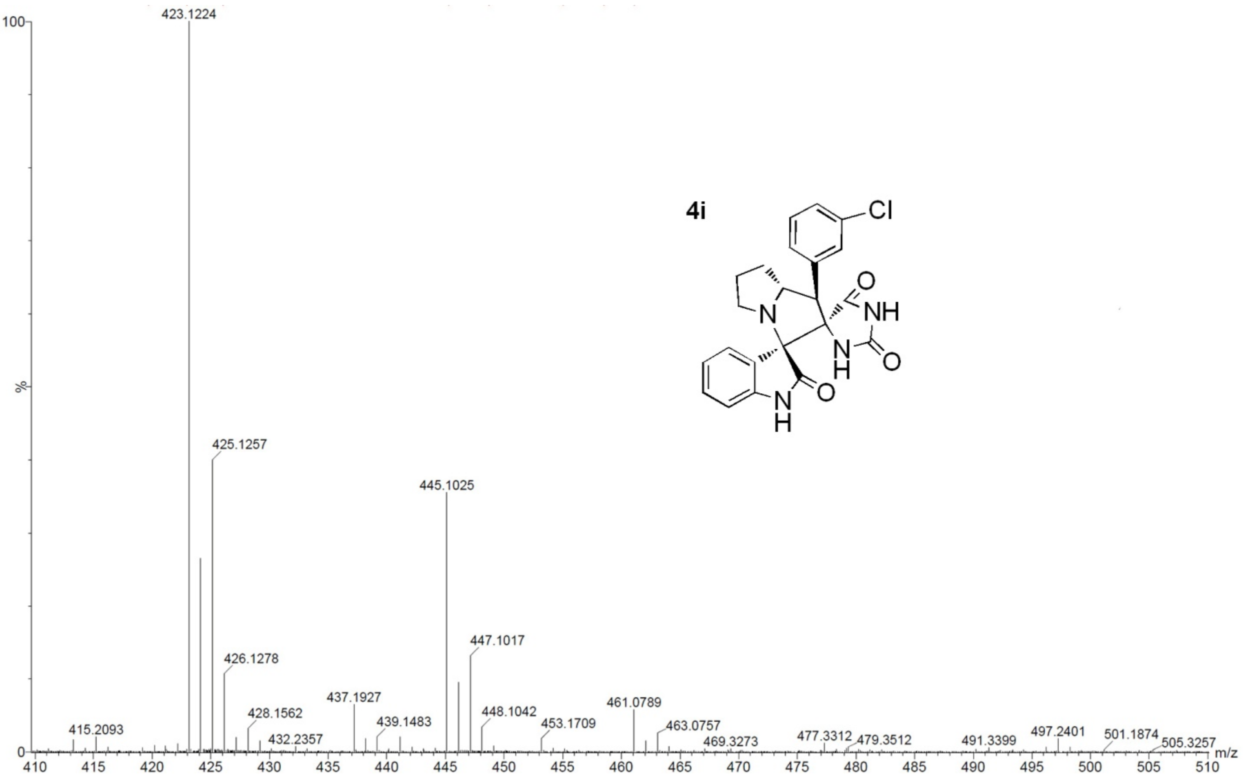

Figure S28. ^{1}H-NMR of Compound **4j**.

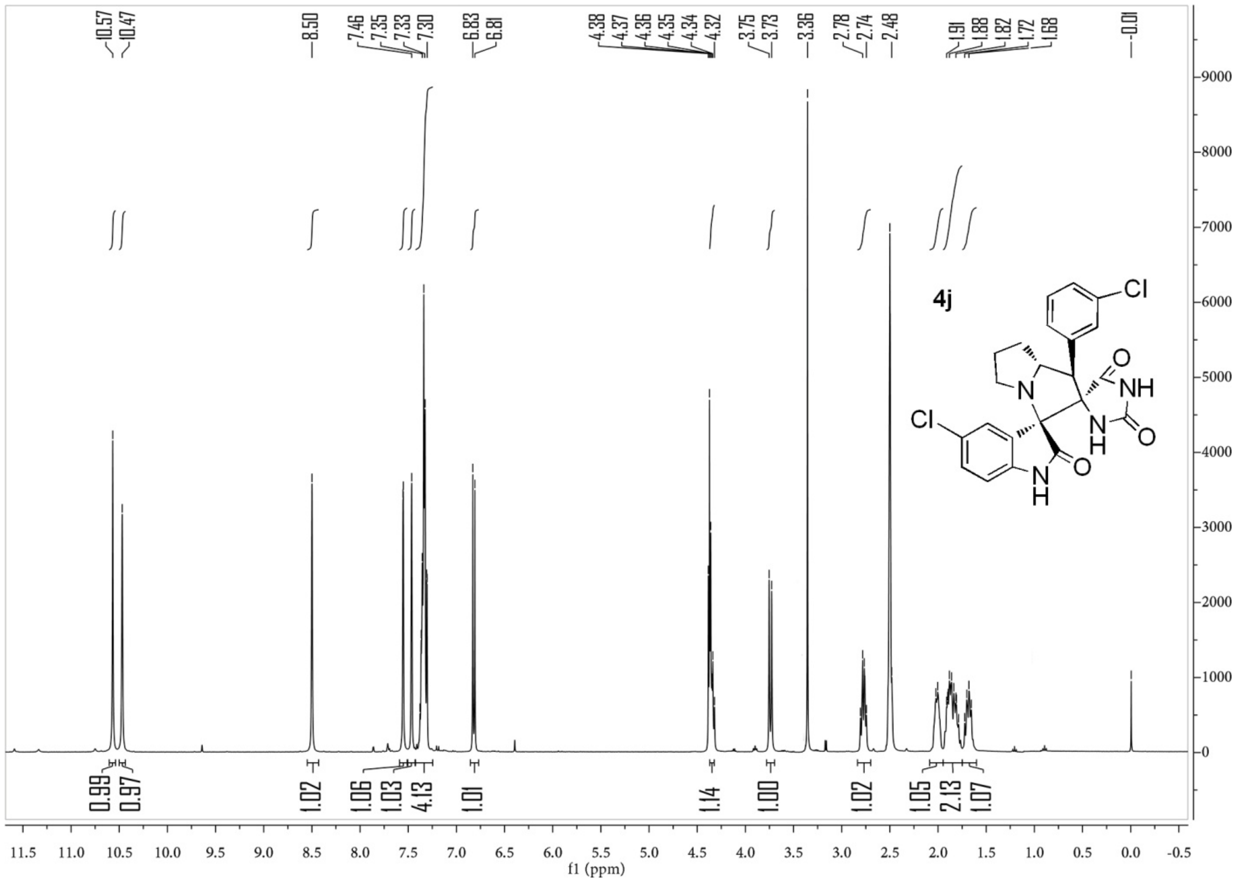

Figure S29. ^{13}C-NMR of Compound **4j**.

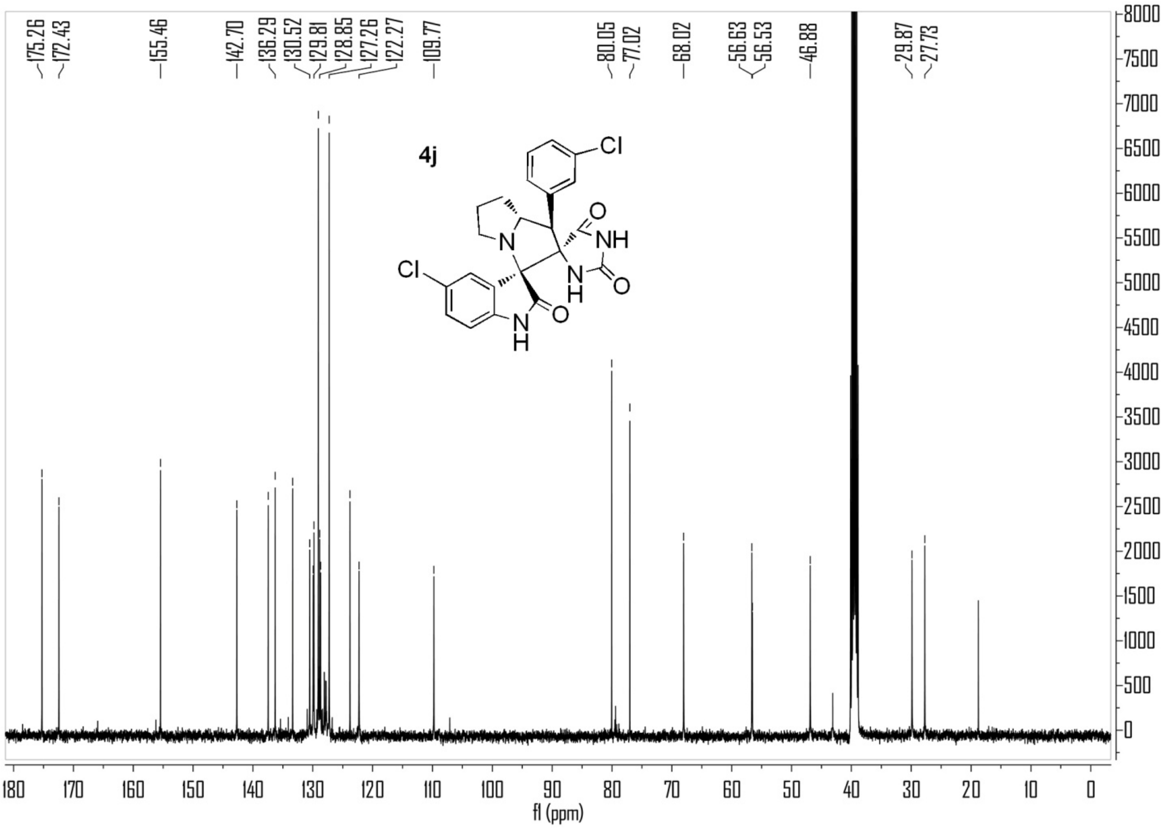

Figure S30. HRMS of Compound **4j**.

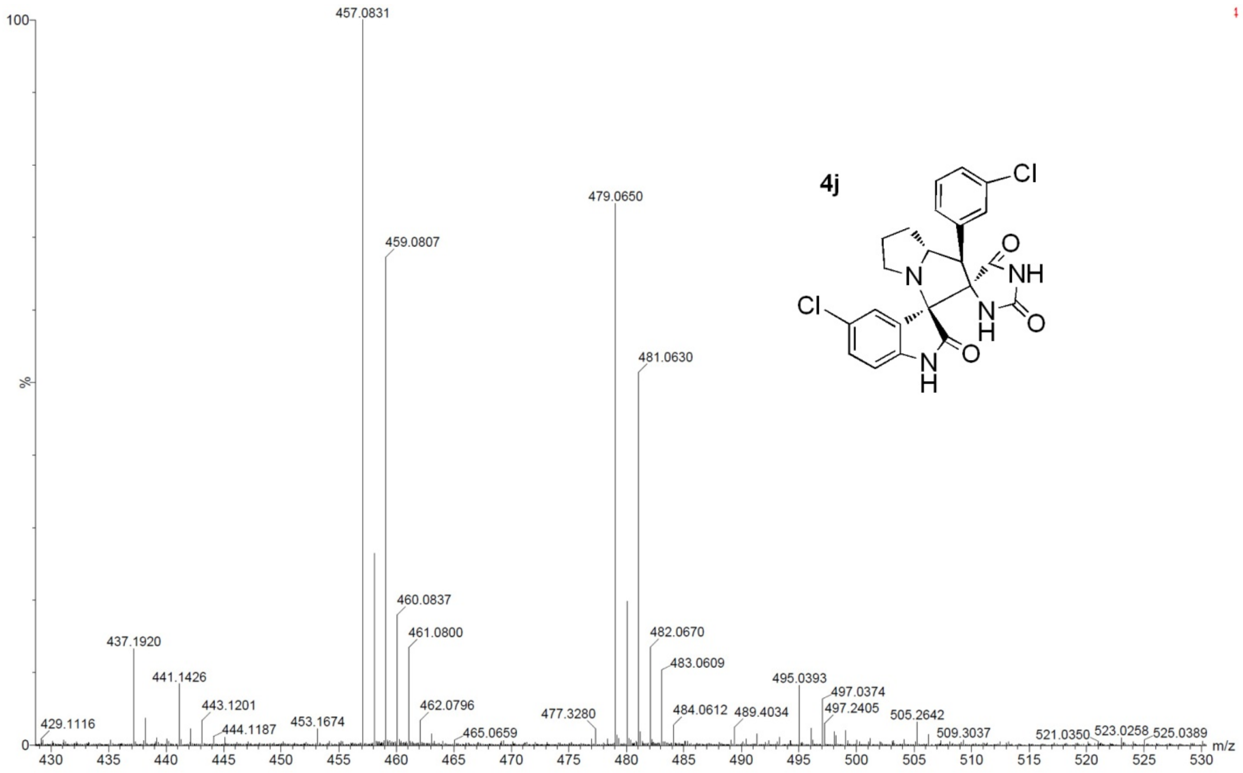

Figure S31. ^{1}H-NMR of Compound **4k**.

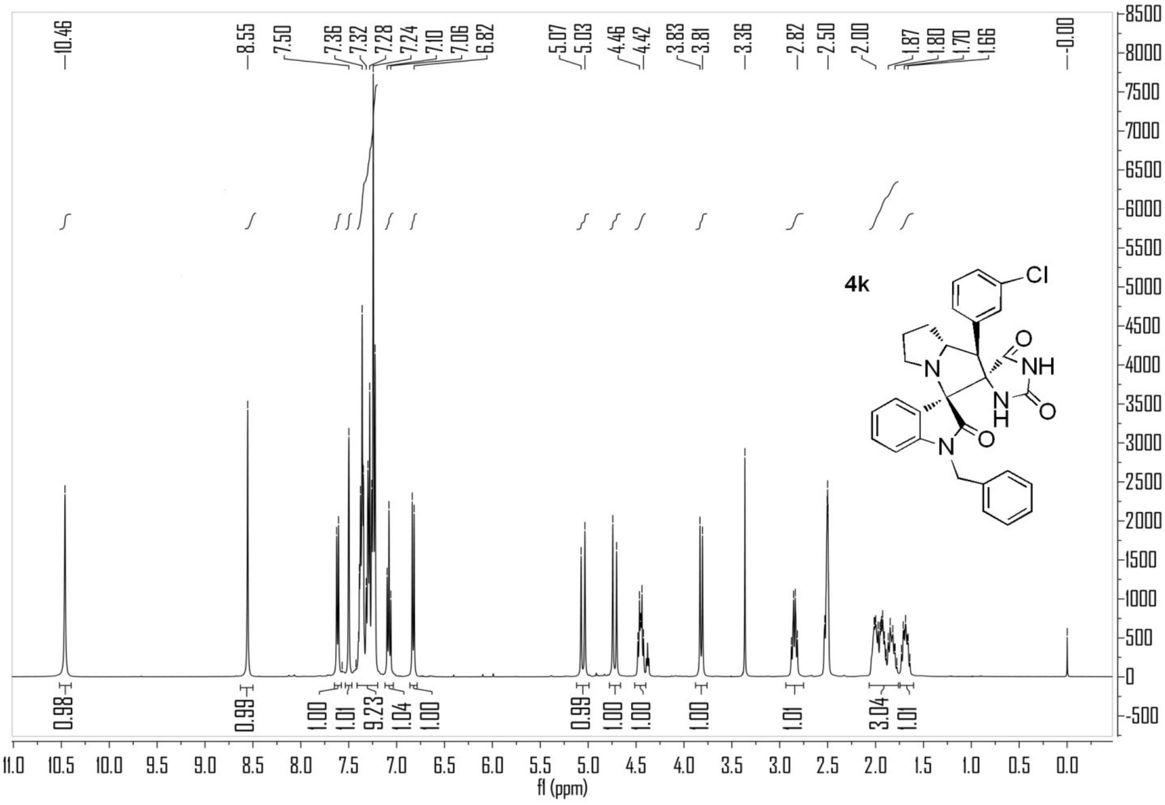

Figure S32. ^{13}C-NMR of Compound **4k**.

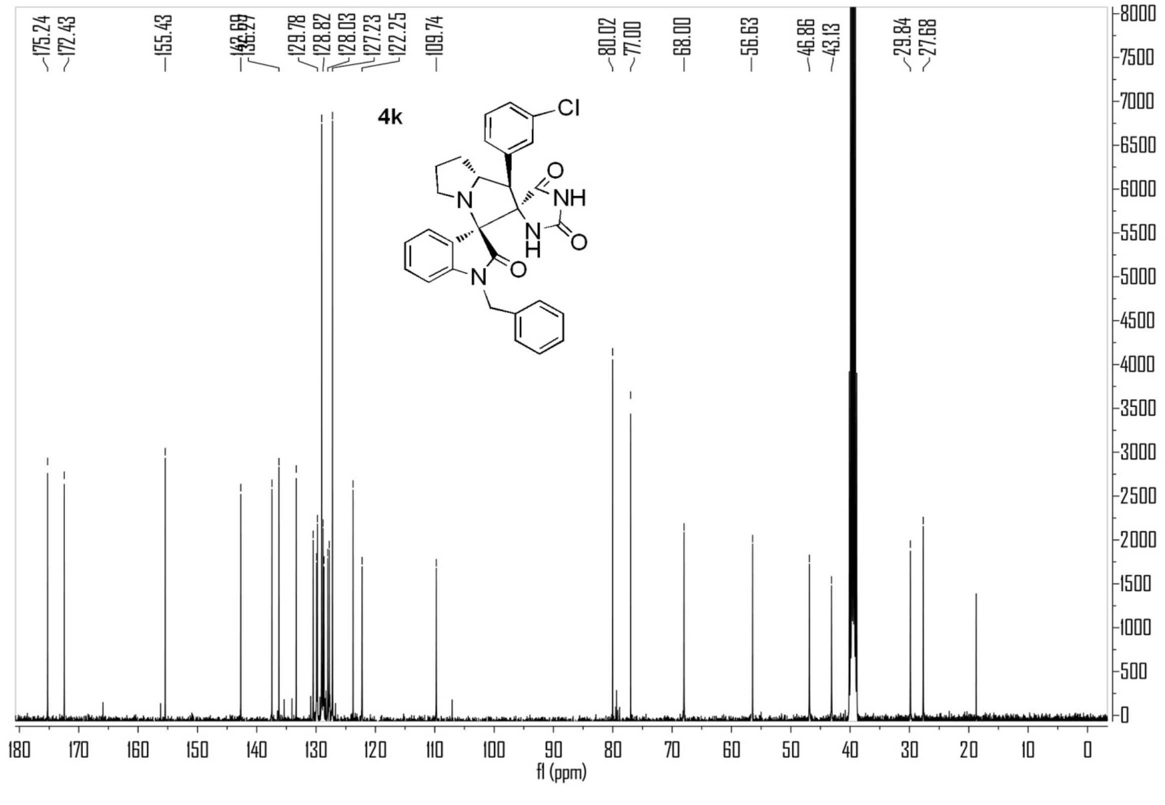

Figure S33. HRMS of Compound **4k**.

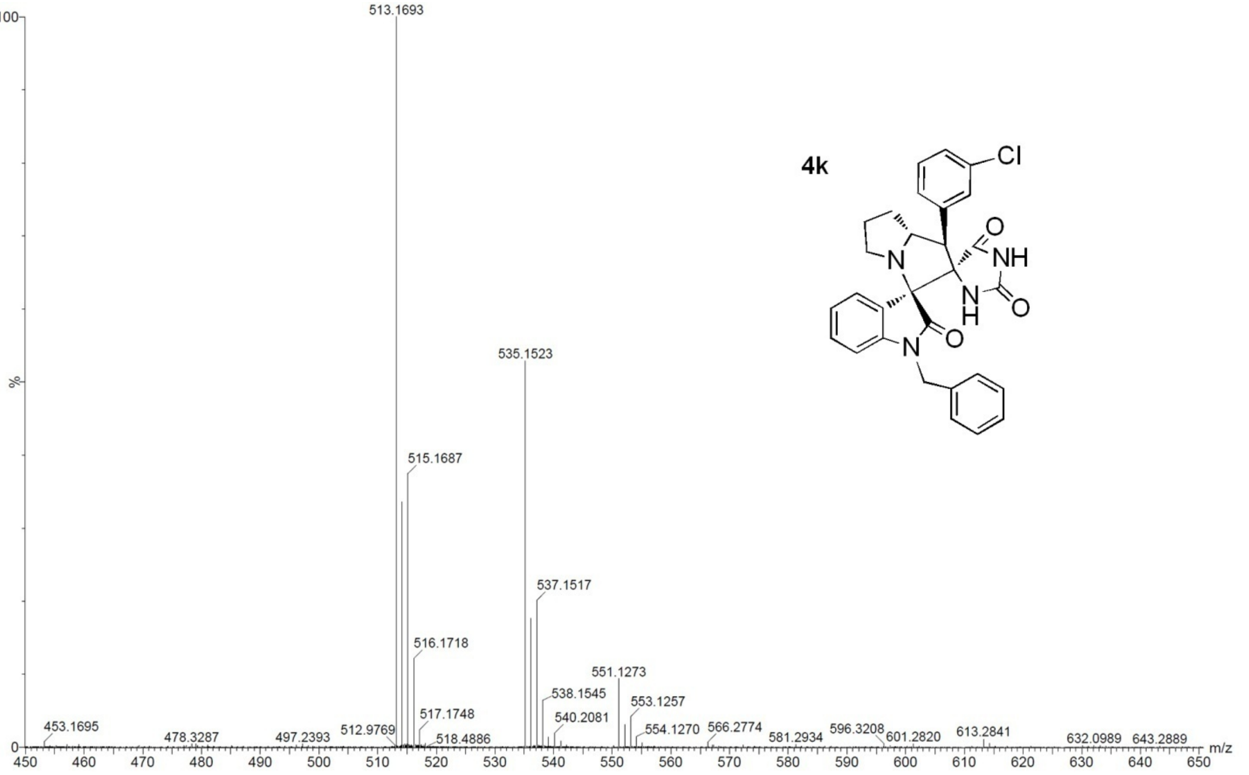

Figure S34. ^{1}H-NMR of Compound **4l**.

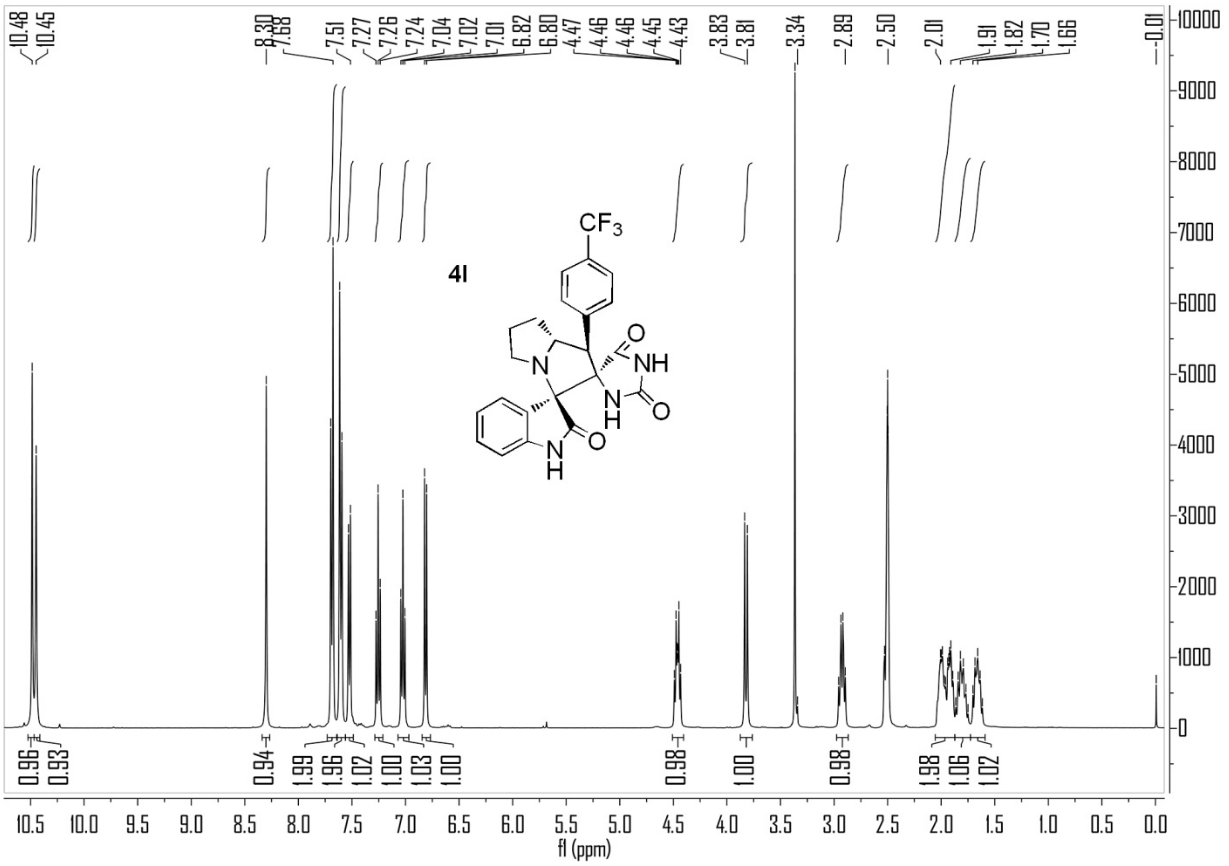

Figure S35. ^{13}C-NMR of Compound **4l**.

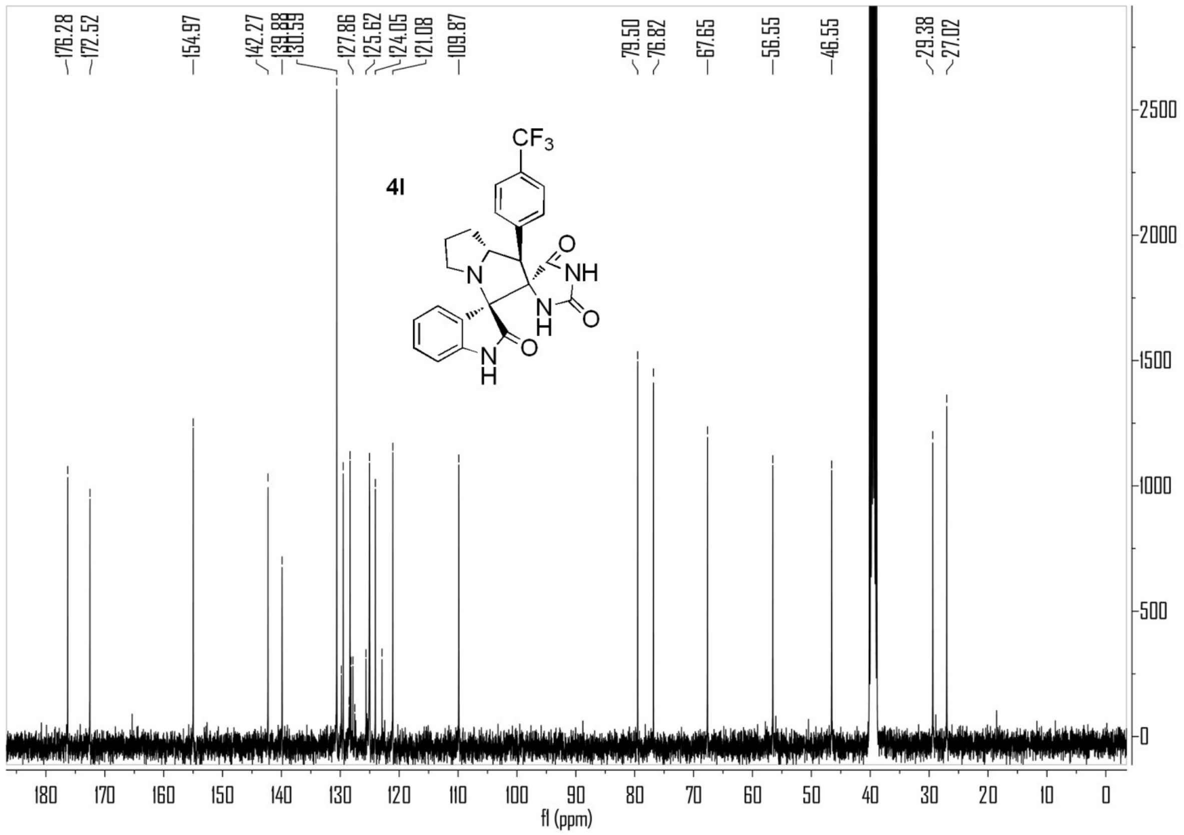

Figure S36. HRMS of Compound **4l**.

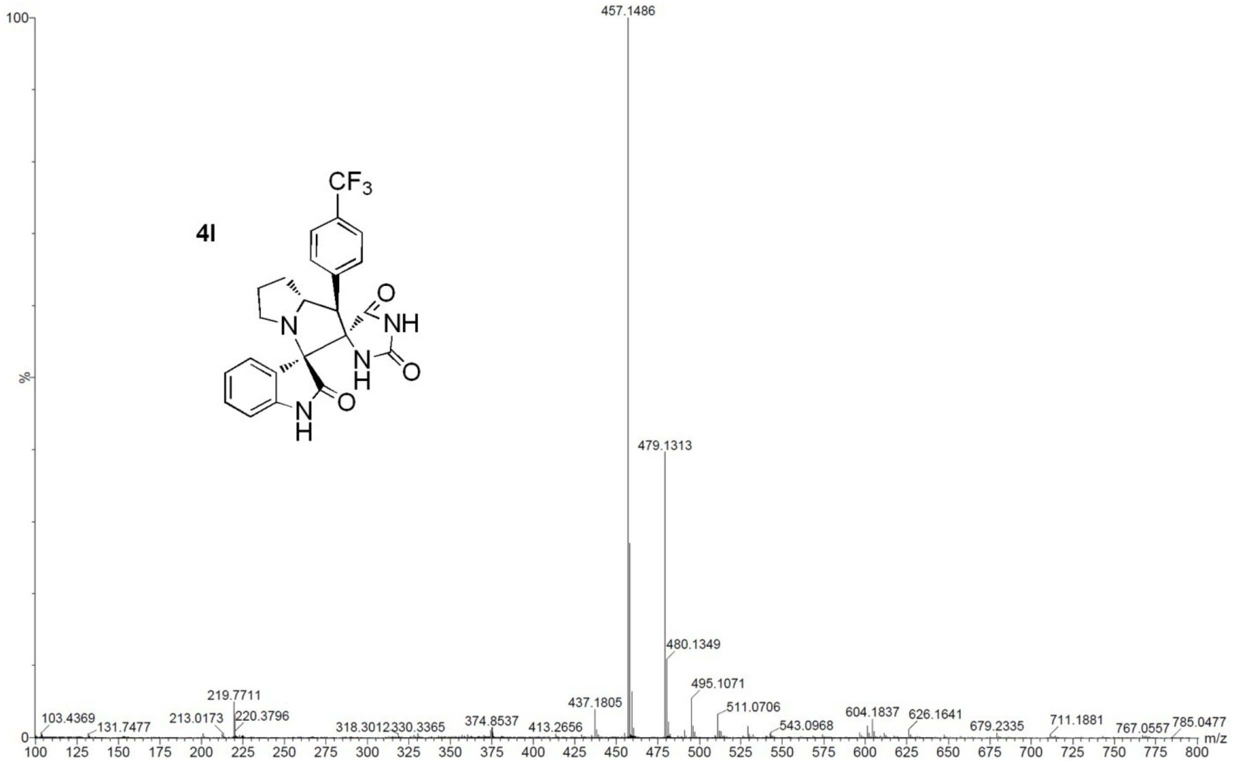

Figure S37. ^{1}H-NMR of Compound **4m**.

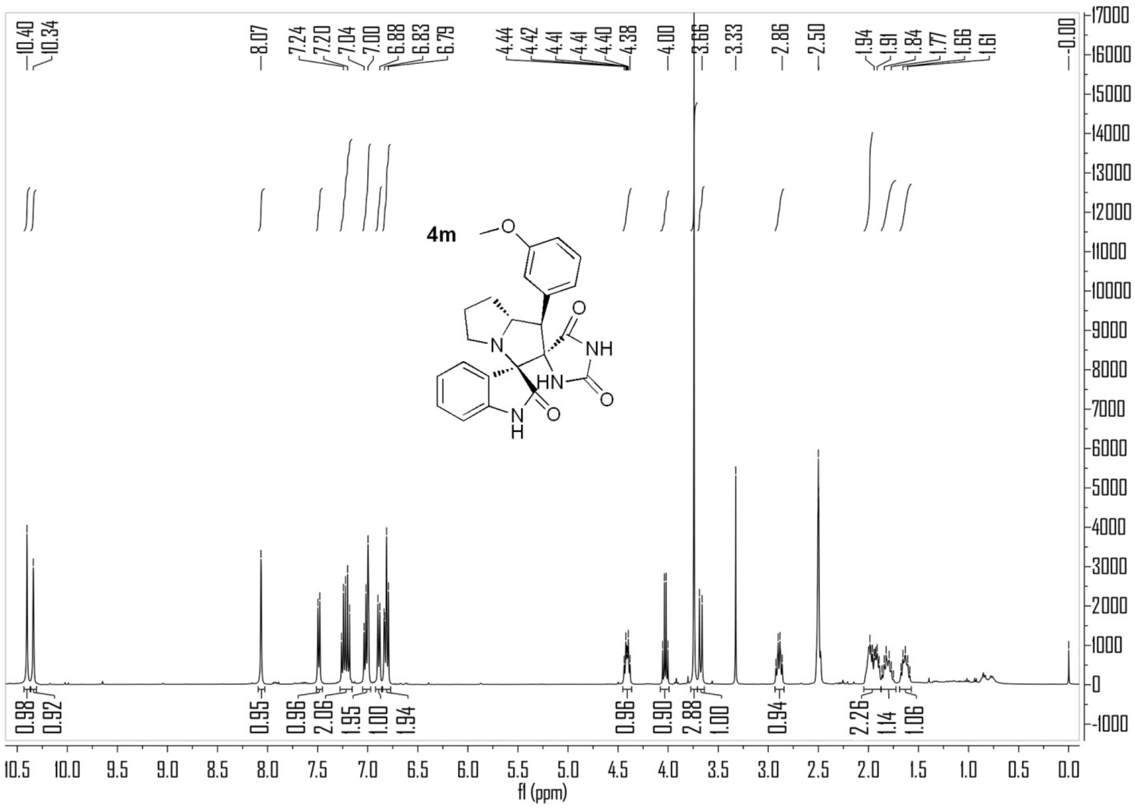

Figure S38. ^{13}C-NMR of Compound **4m**.

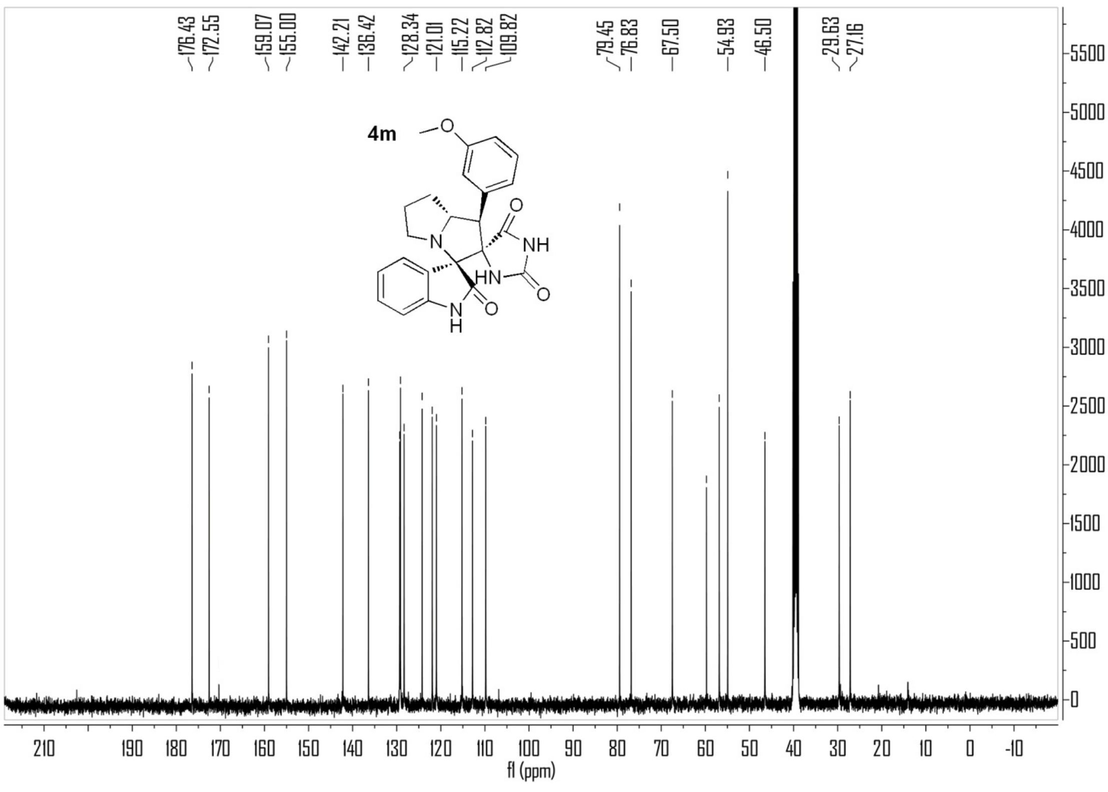

Figure S39. HRMS of Compound **4m**.

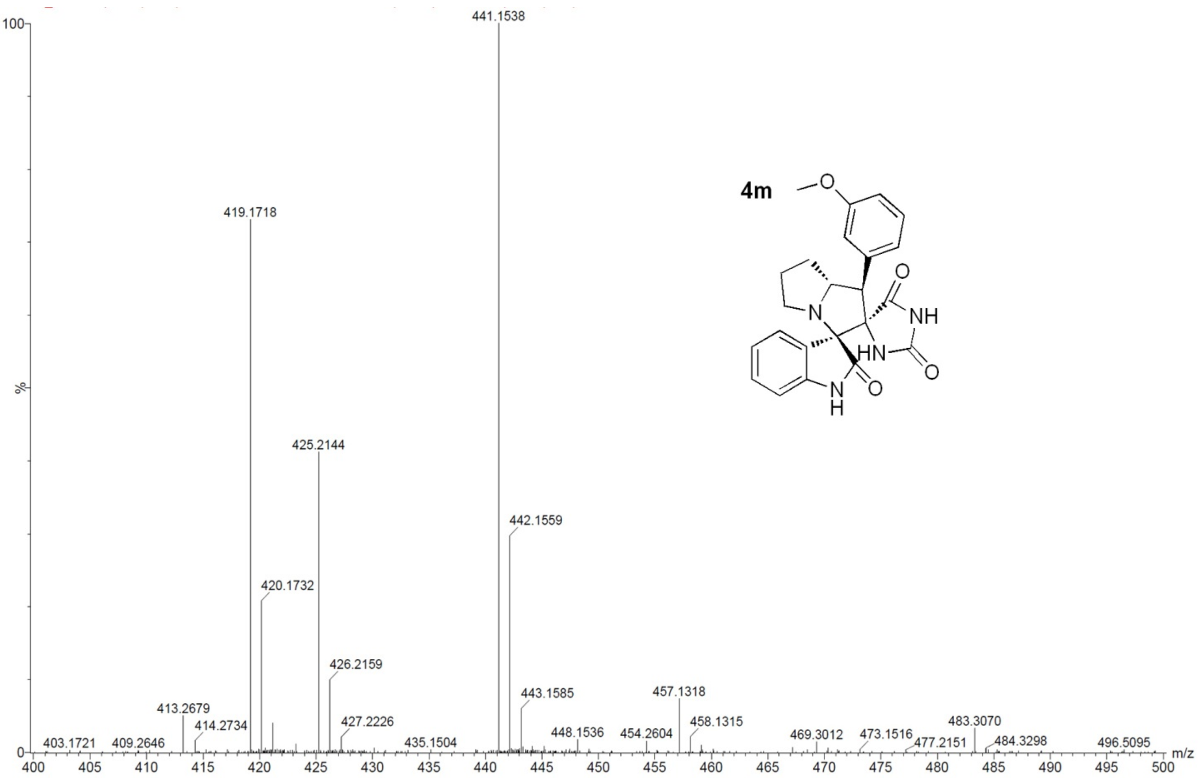

Figure S40. ^{1}H-NMR of Compound **4n**.

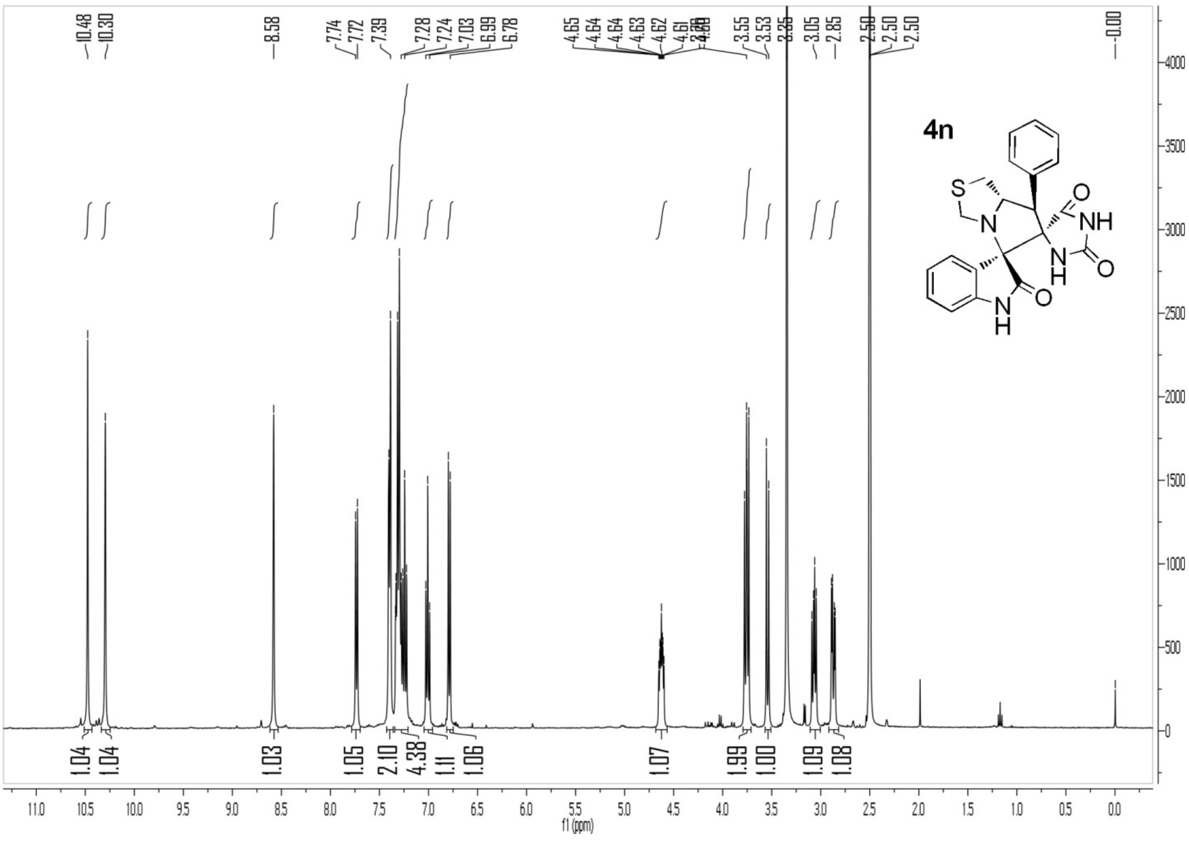

Figure S41. ^{13}C-NMR of Compound **4n**.

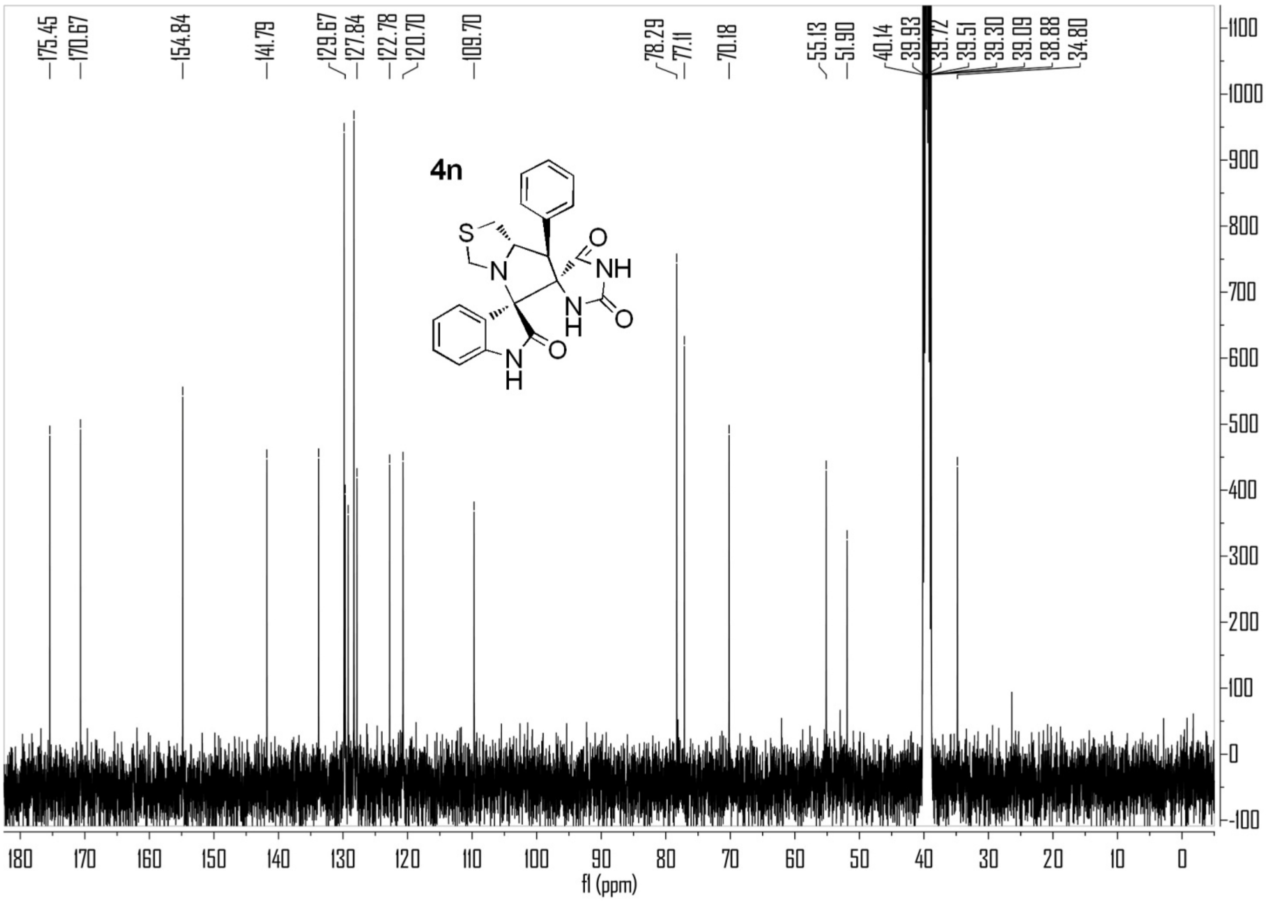

Figure S42. HRMS of Compound **4n**.

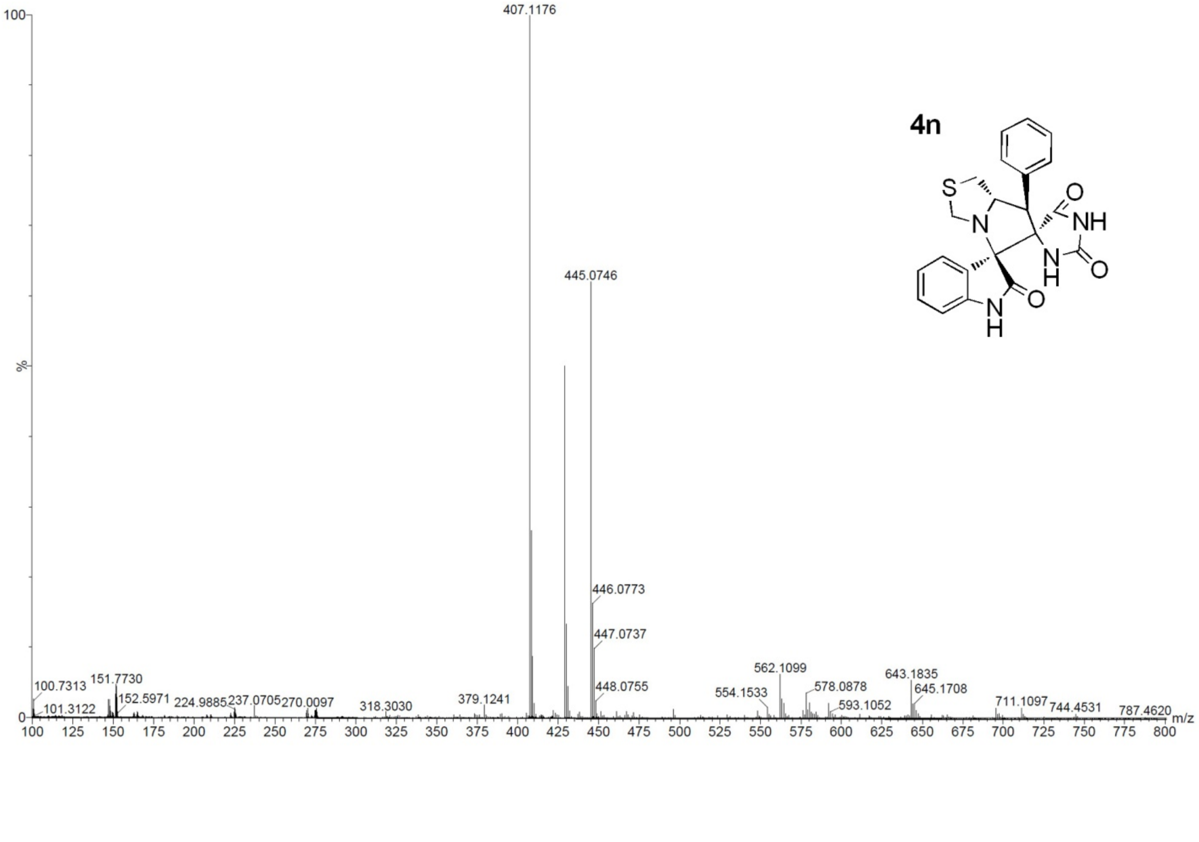

Supplement: Supplementary file 1 [file molecules-18-05142-s001.pdf]
